# Unsupervised and supervised machine learning to identify variability of tumor-educated platelets and association with pan-cancer: A cross-national study

**DOI:** 10.1016/j.fmre.2023.09.004

**Published:** 2023-11-02

**Authors:** Xiong Chen, Runnan Shen, Lin Lv, Dongxi Zhu, Guochang You, Zhenluan Tian, Jinwei Chen, Shen Lin, Jiatang Xu, Guibin Hong, Hu Li, Mingli Luo, Lin Cao, Shaoxu Wu, Kai Huang

**Affiliations:** a Department of Urology Surgery, Sun Yat-sen Memorial Hospital, Sun Yat-sen University, Guangzhou 510120, China; b Zhongshan School of Medicine, Sun Yat-sen University, Guangzhou 510080, China; c Department of Breast Surgery, Sun Yat-sen Memorial Hospital, Sun Yat-sen University, Guangzhou 510120, China; d Department of Urology Surgery, First Affiliated Hospital of Sun Yat-sen University, Guangzhou 510120, China; e Guangzhou Medical University, Guangzhou 511436, China; f Department of Cardiovascular Surgery, Sun Yat-sen Memorial Hospital, Sun Yat-sen University, Guangzhou 510120, China; g Guangdong Provincial Key Laboratory of Malignant Tumor Epigenetics and Gene Regulation, Sun Yat-sen Memorial Hospital, Sun Yat-sen University, Guangzhou 510120, China

**Keywords:** Tumor-educated platelet, Pan-cancer, Platelet transcriptome, Cluster phenotype, Machine learning

## Abstract

Cancer can educate platelets by altering transcriptome profiles. However, the exact education mechanism remains unclear, and the variability of tumor-educated platelet (TEP) transcriptome has not been investigated. In this study, we aimed to build a stratification system for TEP based on machine learning (ML) data-driven patterns and platelet transcriptome profiles. This study included platelet samples from 1,628 cancer participants from European and United States populations, including 18 different and most prevalent types of cancer. Gaussian mixture model (GMM) was used to identify robust clusters and similar education pattern. While extreme gradient boosting (XGBoost) was used to precisely predict the clusters. Three clusters were eventually identified. The cluster results showed robustness and generality, reflected by comparable patterns of important gene expression, cancer type prevalence, and biological annotation across derivation, evaluation and validation cohorts. Cluster 1 (*n* = 346), mainly participated in drug metabolism cytochrome P450, metabolism of xenobiotics by cytochrome P450, and glutathione metabolism. Cluster 2 (*n* = 538) mainly participated in ribosome, spliceosome, and primary immunodeficiency. Cluster 3 (*n* = 744) mainly participated in gap junction and focal adhesion. Based on this novel cluster system, further observational study can investigate the association between these clusters and cancer progression, prognosis, cancer associated thrombosis, treatment resistance (both chemotherapy and immunotherapy), and immune cell infiltration. Overall, in this study, we built the first pan-cancer TEP stratification system based on data-driven patterns of ML and platelet transcriptional profiles. These clusters could help us better understand the variability of the pan-cancer education mechanism.

## Highlights

• The first study to identify variability in tumor-educated platelets.
• Robust variability between the clusters was found based on functional annotation.
• Further research on the association between clusters and cancer progression is needed.

## Graphical abstract

**Figure:**
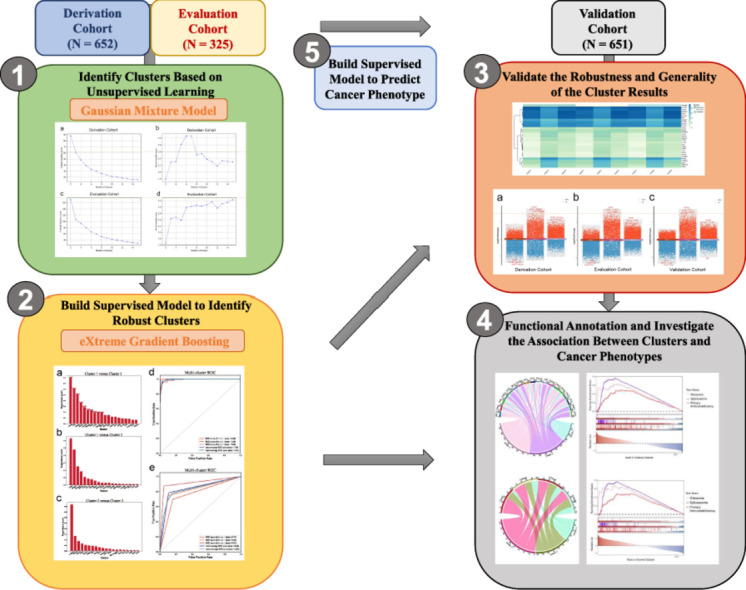

## 1 Introduction

Tumor-educated platelets (TEPs) are functional cells that respond to either local or systemic cues from the tumor parenchyma [1]. Clinical and genetic research has shown that cancer increases the risk of venous thromboembolism [2]. Platelets have been found to participate in the hallmark features of cancer, including metastasis, immune system evasion, neoangiogenesis, and tumor-supportive inflammation [1,3]. In recent decades, TEPs have emerged as a highly specific “liquid biopsy” tool for cancer [1,4]. In't Veld et al. recently employed blood platelet RNA profiles to develop a highly specific pan-cancer blood test covering 18 different tumor types and enabling localization of the primary tumor [5]. However, the variability of the education mechanism, and platelet transcriptome in pan-cancer has not yet been investigated.

Platelet transcriptome profiles can provide information regarding the response to cancer [6]. It is important to use data mining to identify the variability of TEP mRNA since it enables us to find similar educational mechanisms of various cancer phenotypes in each cluster, without requiring excessive classifications for diagnosis [5], [6], [7]. Based on this novel classification or cluster phenotype, we can further investigate the effects of various TEPs on cancer progression and cancer-associated thrombosis (CATs), as well as the potential treatment guidance. Clustering is commonly used to identify subpopulations of patients with gene expression profiles or distinctive genetic variance. To date, the feasibility of clustering has been proven in many studies [8]; however, clustering has not been used to explore the variability of TEPs transcriptome in pan-cancer.

Unsupervised machine learning (ML) is a powerful method for learning from un-labelled datasets, which can identify distinct clusters based on variability within the data [8,9]. As one of unsupervised ML model, gaussian mixture model (GMM) is a powerful parametric cluster algorithm that approximates arbitrary-shaped probability distributions by using multiple gaussian distributions [10]. GMM takes into account multiple cutoff points with confidence (MCPC) and class discovery [11]. Specifically, MCPC discovery refers to calculating the classification thresholds for a variable based on the assigned probabilities of membership to each group, while class discovery involves defining previously unrecognized groups. Supervised learning develops a model based on a training dataset that includes both input features and corresponding labels, which is then used to identify important features and predict new outputs for given inputs accurately [9]. eXtreme gradient boosting (XGBoost) is an efficient, flexible, and highly scalable supervised learning algorithm that uses decision trees as the basic classification and regression models [12]. It utilizes optimization techniques such as gradient boosting algorithm, regularization, parallel processing, and feature processing. As effective tools for analyzing complex biomedical data, GMM has been applied in multiple fields such as gene clustering [10] and quantitative genetics [13], while XGBoost has been widely utilized in disease prediction [12].

Based on a large pan-cancer cohort (1628 individuals) of TEP mRNA, we hypothesized that an unsupervised learning method could help uncover distinct patterns associated with different pathological states. This study aimed to (1) identify clusters based on an unsupervised ML model and investigate the potential bioinformatic status of platelets; (2) train a supervised ML model to distinguish clusters and investigate their associations with cancer phenotypes; and (3) train supervised ML models to distinguish each cancer phenotype and identify the important mRNA features.

## 2 Material and methods

### 2.1 Patients and materials

This study was reanalyzed using raw read counts from GEO: GSE183635 [5]. The GSE183635 study collected platelet samples from 1628 cancer participants from European and United States populations, including 18 different and most prevalent tumor types (including breast cancer [BRCA], cholangiocarcinoma [CHOL], colorectal cancer [CRC], endometrial cancer [ENDO], esophageal cancer [ESO], glioma [GLIO], hepatocellular carcinoma [HCC], head and neck squamous cell carcinoma [HNSSC], lymphoma [LYM], melanoma [MELA], multiple myeloma [MM], non-small cell lung cancer [NSCLC], ovarian cancer [OVCAR], pancreatic ductal adenocarcinoma [PDAC], prostate cancer [PRCA], renal cell carcinoma [RCC], sarcoma [SARC] and urothelial carcinoma [URO]). The entire cohort was subjected to complete randomization and partitioned into three distinct cohorts, the derivation cohort (*n* = 652), evaluation cohort (*n* = 325), and validation cohort (*n* = 651), with proportional ratios of 0.4, 0.2, and 0.4, respectively. The baseline characteristics of patients included in this study are summarized in Table 1.

**Table 1:** **Baseline characteristics of patients included in this study**.

Items	Derivation Cohort	Evaluation Cohort	Validation Cohort	*P*-value
	(n = 652)	(n = 325)	(n = 651)	
Sex (Male)	327 (50.3%)	156 (48.1%)	323 (49.7%)	0.817
Age	62.50 [54.00, 70.00]	65.00 [57.00, 71.00]	63.00 [54.00, 70.00]	0.056
Cancer Type				
BRCA	44 (6.7%)	17 (5.2%)	32 (4.9%)	0.332
CHOL	32 (4.9%)	19 (5.8%)	34 (5.2%)	0.825
CRC	35 (5.4%)	20 (6.2%)	30 (4.6%)	0.579
ENDO	18 (2.8%)	8 (2.5%)	13 (2.0%)	0.664
ESO	5 (0.8%)	2 (0.6%)	8 (1.2%)	0.555
GLIO	54 (8.3%)	24 (7.4%)	54 (8.3%)	0.867
HNSSC	34 (5.2%)	29 (8.9%)	38 (5.8%)	0.068
HCC	10 (1.5%)	3 (0.9%)	10 (1.5%)	0.705
LYM	8 (1.2%)	4 (1.2%)	8 (1.2%)	1
MELA	23 (3.5%)	16 (4.9%)	29 (4.5%)	0.531
MM	12 (1.8%)	5 (1.5%)	14 (2.2%)	0.795
NSCLC	207 (31.7%)	100 (30.8%)	215 (33.0%)	0.757
OVCAR	59 (9.0%)	23 (7.1%)	62 (9.5%)	0.435
PDAC	56 (8.6%)	24 (7.4%)	46 (7.1%)	0.568
PRCA	10 (1.5%)	8 (2.5%)	17 (2.6%)	0.371
RCC	14 (2.1%)	3 (0.9%)	11 (1.7%)	0.381
SARC	17 (2.6%)	14 (4.3%)	22 (3.4%)	0.36
URO	14 (2.1%)	6 (1.8%)	8 (1.2%)	0.435
Cancer Stage				
I Stage	48 (8.8%)	24 (8.5%)	39 (7.1%)	0.571
II Stage	75 (13.8%)	28 (9.9%)	63 (11.5%)	0.238
III Stage	92 (16.9%)	49 (17.4%)	94 (17.2%)	0.984
IV Stage	329 (60.5%)	181 (64.2%)	350 (64.1%)	0.392

Age is represented by a median with interquartile range (IQR) and compared with the Kruskal-Wallis test. Categorical variables are expressed as percentages, compared with chi-square tests. A two-sided *P* < 0.05 is considered statistically significant.

Abbreviation: BRCA, breast cancer; CHOL, cholangiocarcinoma; CRC, colorectal cancer; ENDO, endometrial cancer; ESO, esophageal cancer; GLIO, glioma; HCC, hepatocellular carcinoma; HNSSC, head and neck squamous cell carcinoma; LYM, lymphoma; MELA, melanoma; MM, multiple myeloma; NSCLC, non-small cell lung cancer; OVCAR, ovarian cancer; PDAC, pancreatic ductal adenocarcinomas; PRCA, prostate cancer; RCC, renal cell carcinoma; SARC, sarcoma; URO, urothelial carcinoma.

### 2.2 Methods

#### 2.2.1 Quality control and normalization of the raw read counts data

According to original study, RNA-sequencing reads data of platelets encoded in FASTQ-files were subjected to trimming and clipping of sequence adapters using Trimmomatic (version 0.22), mapped to the human reference genome (hg19) using STAR (version 2.3.0), and summarized using HTseq (version 0.6.1). Then, sample filtering was conducted based on library complexity. Firstly, genes that yielded less than 30 intron-spanning reads in over 90% of the samples across all three cohorts (derivation, evaluation, and validation) were excluded. This filter rule was applied to each cancer type to ensure unique presence of RNAs in each subgroup. Then, samples with fewer than 1500 uniquely detected high confidence genes in each cohort were excluded. Following this, a remove unwanted variation (RUV) correction was systematically applied to the collective cohort, which included the derivation and evaluation cohorts, as well as the validation cohort, to effectively mitigate the influence of potential confounding factors. The confounding factors that needed to be corrected were set based on the library size. And in order to investigate the variability in pan-cancer cohort, the groups were defined as 18 cancer types instead of cancer – non-cancer setting. Next, the median counts-per-million expression value for each gene was estimated via the implementation of a trimmed mean of M-values (TMM) normalization procedure, prior to undergoing log-transformation. At last, a leave-one-sample-out cross-correlation analysis was performed to exclude platelet samples that have low inter-sample correlation (samples with a correlation of less than 0.5 were excluded). The perform.RUVg.correction algorithm and calcNormFactorsThromboseq algorithm, crafted by Best et al., were used to correct and normalize the data [14]. In perform.RUVg.correction algorithm, lib.size was set as variable to assess, and the threshold was set as 0.8.

#### 2.2.2 Development of an unsupervised model for clustering based on the Gaussian mixture model

In this study, GMM was trained using data from the derivation cohort and evaluation cohort. Genes that passed the quality control assessment were included in the model training process. The evaluation cohort was specifically designed to further validate the generality and robustness of the clustering results obtained. To determine the optimal number of clusters that could accurately describe the data, the Calinski–Harabasz (CH) and Davies–Bouldin (DB) scores were utilized. These scores were used as criteria to select the number of clusters that would best fit the data.

#### 2.2.3 Development and validation of a multi-class supervised model for clustering

To further reduce the dependence on multiple features and simplify the model to improve the generalization ability, we initially employed binary-classification extreme gradient boosting (XGBoost) to identify important features that could discriminate between each pair of clusters. Each feature was assigned a weight based on its importance, determined using the information gain criteria. The 30 most important features were then selected based on the sum of their corresponding importance scores across all models. Subsequently, the derivation cohort data was utilized to construct a multi-classification model based on XGBoost, leveraging these 30 features. Patients in the derivation, evaluation and validation cohort were then classified into clusters based on multi-classification XGBoost model. To evaluate the efficacy of this predictive framework in assigning patients to correct clusters, the area under the receiver operating characteristic curve (AUC) was extensively computed via the use of both derivation and evaluation cohort data. This evaluation process involved assessing the consistency between the output prediction results of our supervised model with the ground truth label derived from GMM clustering. Moreover, to provide interpretability of the XGBoost model, the SHapley Additive exPlanations (SHAP) values were calculated for the important features associated with each cluster. Furthermore, to validate the generalizability of the proposed clusters, the association between mRNA features, cancer phenotype and clusters in each cohort were analyzed by drawing heatmaps and circus plots. At last, we also compared the cancer classification score, as derived from the original work of In't Veld et al. across the three clusters.

#### 2.2.4 Development and validation of a supervised model for predicting cancer phenotype

To identify important genes and the potential education mechanisms associated with cancer types and stages, binary-classification models were developed with derivation cohort and validated with evaluation and validation cohort. The specific cancer phenotype (cancer type, e.g., BRCA; cancer stage, e.g., IV stage) was set as positive outcome, while other individuals of the pan-cancer cohort were set as negative controls. Due to an imbalance in the derivation cohort, the synthetic minority oversampling technique (SMOTE) was used to oversample the derivation cohort. SMOTE is an algorithm for resolving the imbalanced class problem by synthesizing new samples and adding them to the derivation cohort, ensuring an equitable balance between the number of positive and negative examples, with a 1:1 ratio achieved. The variance thresholding method was used to reduce the number of features and simplify the models to avoid overfitting. Specifically, out of the initial 5440 features, only those with a variance greater than the defined threshold were selected (the threshold value was 2.5 for predicting cancer stages, while the threshold value was 2.1 for predicting cancer types). To further reduce the number of features, the binary-classification XGBoost models were used to determine the importance of these features, and the top 25 features were selected. Subsequently, the 25-features supervised models were trained with oversampling derivation data and validated separately.

#### 2.2.5 Bioinformatic analysis and functional annotation

To describe the differences in the mRNA profiles of TEPs among different clusters, we performed differential gene expression analysis. A volcano map was plotted to show the results. Based on the fold change ranking of gene expression, gene set enrichment analysis (GSEA) to gain further insights into the biological characteristics of each cluster was performed [15]. The priority-defined set of genes was obtained from C2 catalogs of the MSigDB database (http://www.gsea-msigdb.org/gsea/msigdb/human/collections.jsp#C2), which is based on the Kyoto Encyclopedia of Genes and Genomes (KEGG) pathway. Gene sets with a normalized enrichment score > 1, *P* < 0.05, and false discovery rate (FDR) *q* < 0.25 were considered enriched, and the top three terms were exhibited in our results. To ensure the reliability of our findings, we repeated the aforementioned analysis in all three cohorts.

#### 2.2.6 Post-hoc statistical modeling of potential confounding variables

In order to investigate whether the clusters were influenced by other confounding variables, we followed the post-hoc process of In't Veld et al. The entire pan-cancer cohort was used to perform the post-hoc analysis. Samples with unknown age/or sex status (*n* = 10) were excluded, resulting in a total of 1618 cancer samples used for analysis. The linear models were constructed using the prediction probabilities for each specific cluster, which were derived from the supervised multi-class XGBoost model, as the outcome. A fixed term for the potential confounder (including library size, age, sex, and isolation location), a fixed term for group (the specific cluster) and the interaction between the confounder and group were set as predictors. Subsequently, we calculated and visualized the marginal means for group comparisons and interactions of interest.

#### 2.2.7 Statistical analysis

When comparing the baseline data among cohorts or clusters, categorical variables were expressed as percentages and compared using chi-square tests. Continuous variables were presented as medians with interquartile ranges (IQR) and compared using the Kruskal-Wallis test. Circus and bar chart plots were used to visualize the association between cancer and cluster phenotypes. To investigate the independent association between clusters and cancer phenotypes, logistic regression analysis was performed with/without adjusting for age and sex. A two-sided *P* < 0.05 was defined as statistically significant. Significance tests and visualizations were conducted using R (version:4.2.0). Data preprocessing, model development, as well as further evaluation and validation were conducted using Python (version:3.8.5).

#### 2.2.8 Code availability

The R and Python code for above analysis can be assessed at https://github.com/RNShen/GMM_XGB_TEP_pancancer.

#### 2.2.9 Ethics

This study was a reanalysis of publicly available data and did not require further approval.

## 3 Results and discussion

### 3.1 Results

The flowchart and diagram of this study are presented in Graphic Abstract. The clinical and mRNA data of 1628 cancer patients were randomized into derivation (*n* = 652), evaluation (*n* = 325) and validation (*n* = 651) cohorts, and then analyzed. Firstly, GMM was applied to identify clusters within both the derivation and evaluation cohorts. Subsequently, XGBoost was utilized to construct a supervised model based on pre-defined parameters from the derivation cohort. Next, we applied the supervised model to identify clusters within the derivation, evaluation, and validation cohorts. Finally, the supervised model was utilized to predict each cancer phenotype, aiding in the identification of specific mechanisms underlying cancer development.

The sex, age, cancer type, and cancer stage were not significantly different between the derivation, evaluation and validation cohorts, conforming the randomness of separation (Table 1). The majority of cancer types and stages in three cohorts were NSCLC and IV stage.

Moreover, our utilization of advanced analytical techniques such as GMM and multi-classification XGBoost yielded three highly robust patient clusters that remained consistent across all cohorts. This consistency was further reflected by comparable patterns of important gene expression, cancer type prevalence, and biological annotation. Overall, our results served to reinforce the efficacy of GMM and XGBoost in processing and analysis of complex medical data, as previously introduced.

#### 3.1.1 Identifying clusters based on unsupervised learning

To obtain more robust cluster results, a total of 5440 mRNA features were included in GMM models for clustering analysis in the derivation and evaluation cohort. Based on the CH and DB scores in the two cohorts (Fig. S1 online), we chose *N* = 3 for the final clustering (a higher CH value and lower DB value indicate better clustering). *N* = 3 was found to be in a relatively centered position. Additionally, both the CH and DB results exhibited consistency across the derivation and evaluation cohort, which demonstrated the generality and robustness of the GMM clustering results.

#### 3.1.2 Train supervised ML model to distinguish clusters and validation

**Fig. 1:**
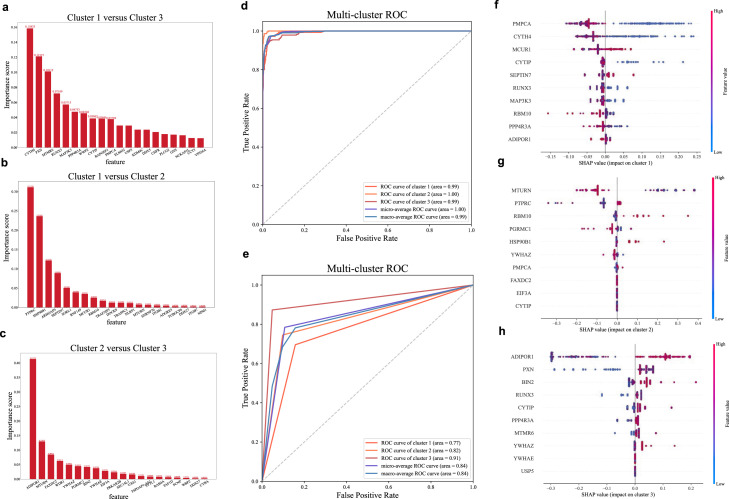
**Predicting clusters based on unsupervised and supervised models.** GMM was used to identify the variability of TEP based on 5440 mRNA features. Multi-class XGBoost was used to train supervised model based on Top 30 features. (a-c) The importance rank of features of 3 models built for distinguishing between each pair of clusters. XGBoost model was used to select features. For calculating the feature importance, we used the feature_importances_property of XGBClassifier, and the calculation method was based on "gain", which calculates the average gain across all splits for each feature; (d) ROC curves of multi-class XGBoost model in derivation cohort; (e) ROC curves of multi-class XGBoost model in evaluation cohort, which were obtained by comparing prediction output results of multi-class XGBoost and the ground truth obtained by GMM; (f-h) SHAP value of top 10 mRNA features associated with cluster 1–3, identified by XGBoost model. Abbreviation: ROC, receiver operating characteristic; GMM, gaussian mixture model; TEP, tumor-educated platelets; XGBoost, extreme gradient boosting; SHAP, Shapley Additive exPlanations.

**Fig. 2:**
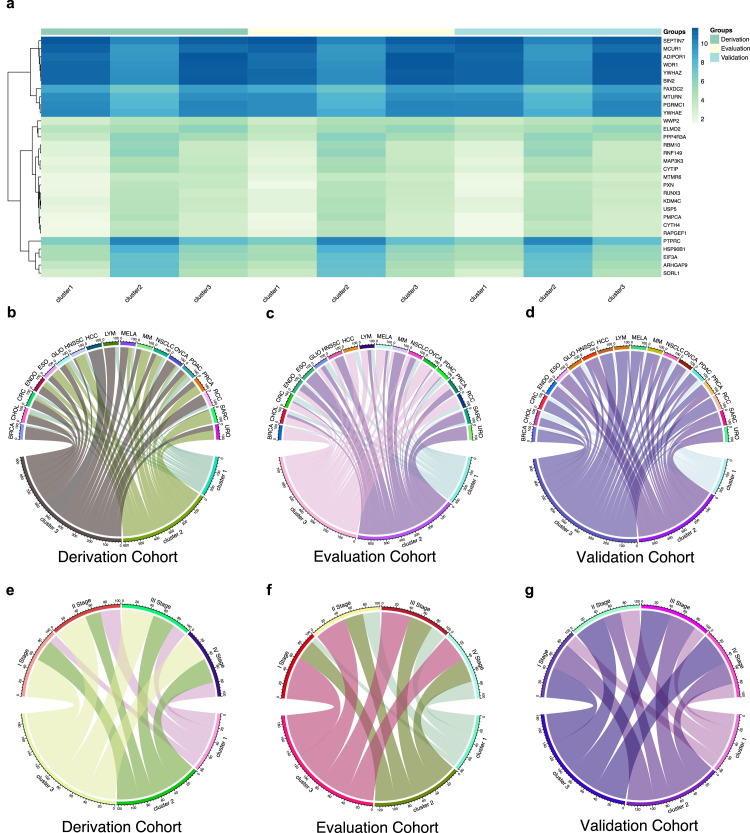
**Predicting clusters based on supervised model and association between clusters and pan-cancer phenotype across derivation, evaluation, and validation cohort.** Multi-class XGBoost was used to train supervised model based on Top 30 features. (a) Heatmap of expression of Top 30 mRNA features in three clusters in derivation, evaluation, and validation cohort. (b-d) The association between cancer types and the three clusters identified with a supervised model in the derivation, evaluation, and validation cohorts are shown in the circus graph. The upper part of the graph represents the percentage of clusters in each cancer type. (e-g) The association between cancer stages and the three clusters identified with a supervised model in the derivation, evaluation, and validation cohorts are shown in the circus graph. The upper part of the graph represents the percentage of clusters in each cancer stage. Abbreviation: BRCA, breast cancer; CHOL, cholangiocarcinoma; CRC, colorectal cancer; ENDO, endometrial cancer; ESO, esophageal cancer; GLIO, glioma; HCC, hepatocellular carcinoma; HNSSC, head and neck squamous cell carcinoma; LYM, lymphoma; MELA, melanoma; MM, multiple myeloma; NSCLC, non-small cell lung cancer; OVCAR, ovarian cancer; PDAC, pancreatic ductal adenocarcinomas; PRCA, prostate cancer; RCC, renal cell carcinoma; SARC, sarcoma; URO, urothelial carcinoma; XGBoost, extreme gradient boosting.

The importance ranking of the features of the three models used to distinguish between the two cluster phenotypes (Cluster 1 vs Cluster 2, Cluster 1 vs Cluster 3, Cluster 2 vs Cluster 3) is shown in Fig. 1a-c. Finally, the 30 most important weighted features (MAP3K3, YWHAZ, PGRMC1, CYTIP, ELMO2, KDM4C, HSP90B1, MTURN, YWHAE, USP5, RUNX3, BIN2, EIF3A, ADIPOR1, MCUR1, RBM10, CYTH4, PXN, PTPRC, SEPTIN7, WWP2, RNF149, PMPCA, PPP4R3A, WDR1, MTMR6, ARHGAP9, RAPGEF1, SORL1, FAXDC2) were included in the XGBoost model to train the multi-classification model. The AUC and ROC were used to show the discrimination ability of the supervised ML model (Fig. 1d-e). The final model achieved a micro-average AUC of 1.00 and a macro-average AUC of 0.99 in derivation cohort, a micro-average AUC of 0.84 and a macro-average AUC of 0.84 in evaluation cohort. The SHAP values for different cluster phenotypes are shown in Fig. 1f-h. In our multi-classification XGBoost model, the features with the greatest impact on Cluster 1 were PMPCA, CYTH4 and MCUR1, while for Cluster 2 were MTURN, PTPRC and RBM10, for Cluster 3 were ADIPO1, PXN and BIN2. Subsequently, three clusters in derivation, evaluation and validation cohort were predicted with supervised model respectively. The heatmap in Fig. 2a shows the expression of the 30 mRNA features stratified by clusters in the three cohorts. The clusters in the evaluation and validation cohorts had mRNA features similar to those in the derivation cohort. The above results demonstrated the good discrimination ability of supervised ML model and the generality of the clustering results.

#### 3.1.3 Association between clusters and cancer phenotypes

The baseline characteristics of patients in different clusters in three cohorts are summarized in Tables S1–3 (online). Cluster 1 had the lowest number across three cohorts (*n* = 346 in total), and was relatively challenging to predict (AUC = 0.77 in evaluation cohort, Fig. 1e). The cancer type presented similar prevalence pattern across three cohorts in Cluster 1. The top 3 cancer types were NSCLC (36.8%, 42.1% and 42.5% in derivation, evaluation and validation cohorts), GLIO (11.8%, 11.8% and 11.2% in three cohorts) and PDAC (14.0%, 11.8% and 10.4% in three cohorts). In terms of cancer stage, Cluster 1 had a lower percentage of I stage (5.1%, 3.0%, and 6.2% in three cohorts) and a higher percentage of II stage (18.8%, 10.6%, and 16.1% in three cohorts).

Cluster 2 had an intermediate number in both cohort (*n* = 538 in total) and could be predicted well (AUC = 0.82 in evaluation cohort, Fig. 1e). The prevalence pattern of cancer types was similar across the three cohorts in Cluster 2. The top 3 cancer types were NSCLC (43.3%, 36.3% and 40.4% in three cohorts), HNSSC (10.6%, 16.8% and 13.0% in three cohorts) and SARC (7.4%, 9.7% and 7.7% in three cohorts). In terms of cancer stage, Cluster 2 had lower percentage of II stage (9.4%, 8.4%, and 8.8% in three cohorts).

Cluster 3 had the highest number in both cohort (*n* = 744 in total) and could be predicted well (AUC = 0.91 in evaluation cohort, Fig. 1e). The cancer type presented similar prevalence pattern across three cohorts in Cluster 3. The top 3 cancer types were NSCLC (21.1%, 19.9% and 23.9% in three cohorts), OVCAR (13.7%, 14.0% and 10.4% in three cohorts) and BRCA (13.0%, 11.0% and 8.7% in three cohorts). When compared between clusters, BRCA, HNSSC, NSCLC, SARC and URO all showed significant differences in three cohorts (*P* < 0.05). However, cancer stage didn't show significant results.

In the circus graph and the bar chat of clusters in three cohorts (Fig. 2b-g, Figs. S2-S3 online), which showed the percentage of clusters in each cancer phenotypes, we found that BRCA (*n* = 93), CRC (*n* = 85), HCC (*n* = 23), LYM (*n* = 20), MELA (*n* = 68), OVCAR (*n* = 144), RCC (*n* = 28) were predominantly comprised with Cluster 3 (The percentages for Cluster 3 in above cancer types in total cohort were 87.1%, 63.5%, 91.3%, 75%, 66.2%, 63.9%, and 82.1%). While ESO (*n* = 15), HNSSC (*n* = 101), SARC (*n* = 53) and URO (*n* = 28) were predominantly comprised with Cluster 2 (The percentages for Cluster 2 in above cancer types in total cohort were 80.0%, 68.3%, 81.1%, and 78.6%). CHOL (*n* = 85), PDAC (*n* = 126) were predominantly comprised with Cluster 3 and Cluster 1 (The percentages for Cluster 3 and 1 in CHOL in total cohort were 55.3% and 34.1%, while in PDAC were 45.2% and 33.3%). ENDO (*n* = 39), MM (*n* = 31), PRCA (*n* = 35) were predominantly comprised with Cluster 3 and Cluster 2 (The percentages for Cluster 3 and 2 in ENDO in total cohort were 48.7% and 38.5%, while in MM were 41.9% and 54.8%, in PRCA were 48.6% and 51.4%). Notably, HCC was not observed to be present within Cluster 2, whereas URO and PRCA was notably absent from Cluster 1. Two large cohorts of cancer, GLIO (*n* = 132) and NSCLC (*n* = 522), might suggest different education mechanism compared to the above cancer types. GLIO and NSCLC exhibited a relatively balanced presence across all three clusters. However, GLIO was predominantly comprised with Cluster 3 (43.2%) and NSCLC was predominantly comprised with Cluster 2 (42.0%). In comparison of cancer classification score, we found that Cluster 3 had a lower score, but its underlying mechanism remains unclear and requires further investigation (Table S4, Fig. S4 online).

In the multi-variable adjusted logistic regression analysis, Cluster 3 was found to be an independent significant prediction factor for BRCA, GLIO, NSCLC, OVCAR. While Cluster 2 was independent significant prediction factor for CHOL, CRC, HNSSC, PDAC, SARC and II stage (Table S5, online).

The above results indicated that our cluster results in each cohort had a similar pattern of cancer type prevalence. It suggests that different types of cancer may share similar education mechanisms.

#### 3.1.4 Functional annotation in each cluster phenotype

**Fig. 3:**
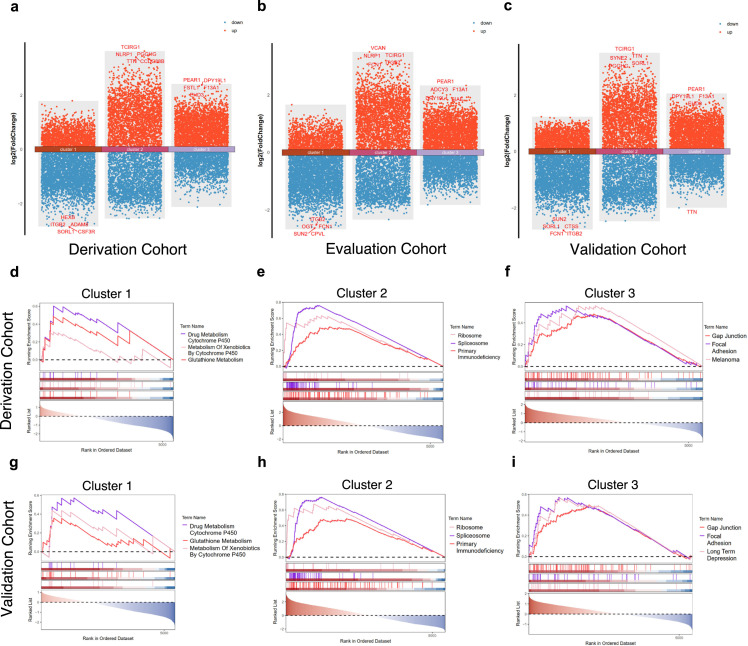
**Differentially expression genes in derivation, evaluation, and validation cohort, and functional annotation of 3 clusters in both derivation and validation cohort.** (a-c) Volcano plot shows up and down regulated genes (compared to other clusters) in each cluster in derivation, evaluation, and validation cohort; (d-f) GSEA of differentially expression genes of cluster 1–3 in derivation cohort; (g-i) GSEA of differentially expression genes of cluster 1–3 in validation cohort. Abbreviation: GSEA, gene set enrichment analysis.

The differentially expressed genes (DEG) in each cluster in three cohorts are shown in volcano plots, and the top five DEG (according to fold change) are labeled (Fig. 3a–c). For instance, DEG in Cluster 1 represents the comparison between Cluster 1 and the combined Cluster 2 and 3. The results reveal that the clusters in the derivation cohort had a DEG pattern similar to that of the evaluation and validation cohort. Based on GSEA and KEGG, the functional annotations of each cluster in the derivation and validation cohort are shown in Fig. 3d–i, while that for the evaluation cohort is shown in Fig. S5 (online). Cluster 1 is primarily involved in drug metabolism cytochrome P450, metabolism of xenobiotics by cytochrome P450 and glutathione metabolism. Cluster 2 is mainly associated with ribosome, spliceosome and primary immunodeficiency. Cluster 3 mainly participated in gap junction, focal adhesion. Our findings were highly reproducible across all three cohorts, demonstrating remarkable consistency and reliability of biology status of each cluster. The core enrichment genes of the top 3 KEGG pathways in each cluster in derivation cohort are shown in Table S6 (online).

Furthermore, to investigate which cluster might contribute to CATs, we estimated the expression pattern of genes involved in platelet activation and complement and coagulation cascades in the three clusters (Fig. S6 online). We found that Cluster 2 displayed a distinct expression pattern compared to Cluster 1 and 3 in this context. In Cluster 1 and 3, genes such as ITGA2B, GNAS, ITGB1, PTGS1, VWF, PROS1, and F13A1 were found to be up-regulated, whereas genes like ITGB2, SERPINA1, and ITGAX were down-regulated, relative to Cluster 2. However, it is important to note that further validation is necessary to fully characterize their biological significance.

#### 3.1.5 Supervised model to predict cancer phenotype and identify important mRNA features

Twenty-three models based on XGBoost were built to predict different cancer types and stages, with the AUCs of the models in the derivation, evaluation and validation cohorts presented in Table 2. For cancer types, the models predicting BRCA, ENDO, GLIO, HCC, HNSSC, MELA, MM, NSCLC, OVCAR, PRCA, RCC, SARC and URO exhibited good discrimination ability (AUC > 0.7). Although the AUC of models that predicted stages II and III tumor were lower than 0.7, the models still had good results in distinguishing advanced stage IV tumor and early stage I tumor. This result was consistent with that of the original publication [5]. The top three important genes are listed in Table 3. And the functional annotation of 25 variables in each model based on KEGG analysis is shown in Table 3. For example, genes predicting NSCLC mainly participated in mTOR signaling pathway, while genes predicting OVCAR mainly participated in IL-17 signaling pathway. Through the use of a supervised model, we were able to effectively identify specific education mechanisms underlying different cancer types and stages, with the identified important genes also capable of further predicting specific cancer phenotypes.

**Table 2:** **AUC of supervised model for predicting different cancer phenotypes in different cohorts**.

Predicting Outcome	Derivation Cohort		Evaluation Cohort		Validation Cohort	
	AUC of Supervised Model #	Main Cluster 2	AUC of Supervised Model #	Main Cluster 2	AUC of Supervised Model #	Main Cluster 2
	(95% CI)		(95% CI)		(95% CI)	
Cancer Type						
BRCA	0.99 (0.99-1.00)	Cluster 3	0.92 (0.86-0.97)	Cluster 3	0.84 (0.78-0.91)	Cluster 3
CHOL	1.00 (0.99-1.00)	Cluster 3	0.67 (0.58-0.76)	Cluster 3	0.55 (0.47-0.64)	Cluster 3
CRC	0.99 (0.99-0.99)	Cluster 3	0.57 (0.47-0.67)	Cluster 3	0.6 (0.51-0.69)	Cluster 3
ENDO	1.00 (1.00-1.00)	Cluster 3	0.97 (0.95-0.99)	Cluster 2	0.84 (0.74-0.93)	Cluster 3
ESO	1.00 (1.00-1.00)	Cluster 2	0.67 (0.48-0.86)	Cluster 2	0.49 (0.33-0.65)	Cluster 2
GLIO	1.00 (0.99-1.00)	Cluster 3	0.88 (0.81-0.94)	Cluster 1	0.83 (0.78-0.88)	Cluster 3
HCC	1.00 (1.00-1.00)	Cluster 3	0.83 (0.56-0.99)	Cluster 3	0.82 (0.67-0.94)	Cluster 3
HNSSC	1.00 (1.00-1.00)	Cluster 2	0.82 (0.74-0.90)	Cluster 2	0.83 (0.77-0.89)	Cluster 2
LYM	1.00 (1.00-1.00)	Cluster 3	0.57 (0.37-0.76)	Cluster 3	0.57 (0.41-0.76)	Cluster 3
MELA	1.00 (1.00-1.00)	Cluster 3	0.72 (0.63-0.80)	Cluster 3	0.77 (0.69-0.84)	Cluster 3
MM	1.00 (1.00-1.00)	Cluster 2	0.94 (0.86-0.98)	Cluster 2	0.77 (0.67-0.87)	Cluster 3
NSCLC	0.93 (0.92-0.94)	Cluster 2	0.77 (0.72-0.81)	Cluster 2	0.78 (0.75-0.81)	Cluster 2
OVCAR	1.0 (0.99-1.00)	Cluster 3	0.91 (0.87-0.94)	Cluster 3	0.85 (0.81-0.89)	Cluster 3
PDAC	0.99 (0.98-0.99)	Cluster 3	0.62 (0.53-0.72)	Cluster 3	0.66 (0.59-0.72)	Cluster 3
PRCA	1.0 (1.00-1.00)	Cluster 2	0.86 (0.77-0.95)	Cluster 2/3	0.85 (0.76-0.94)	Cluster 3
RCC	1.00 (1.00-1.00)	Cluster 3	0.87 (0.77-0.98)	Cluster 3	0.85 (0.75-0.92)	Cluster 3
SARC	1.00 (1.00-1.00)	Cluster 2	0.77 (0.64-0.89)	Cluster 2	0.77 (0.66-0.87)	Cluster 2
URO	1.0 (1.00-1.00)	Cluster 2	0.84 (0.75-0.92)	Cluster 2	0.93 (0.84-0.99)	Cluster 2
Cancer Stage						
I Stage	0.99 (0.99-0.99)	Cluster 3	0.76 (0.68-0.83)	Cluster 3	0.77 (0.69-0.84)	Cluster 3
II Stage	0.96 (0.95-0.97)	Cluster 3	0.46 (0.37-0.55)	Cluster 3	0.57 (0.50-0.63)	Cluster 3
III Stage	0.96 (0.95-0.96)	Cluster 3	0.49 (0.42-0.57)	Cluster 3	0.56 (0.51-0.61)	Cluster 3
IV Stage	0.88 (0.86-0.90)	Cluster 3	0.66 (0.61-0.71)	Cluster 3	0.72 (0.69-0.76)	Cluster 3
Early Cancer (I + II Stage)	0.95 (0.94-0.96)	Cluster 3	0.70 (0.63-0.76)	Cluster 3	0.69 (0.64-0.74)	Cluster 3

Abbreviation: AUC, area under the curve; CI, confidence interval; BRCA, breast cancer; CHOL, cholangiocarcinoma; CRC, colorectal cancer; ENDO, endometrial cancer; ESO, esophageal cancer; GLIO, glioma; HCC, hepatocellular carcinoma; HNSSC, head and neck squamous cell carcinoma; LYM, lymphoma; MELA, melanoma; MM, multiple myeloma; NSCLC, non-small cell lung cancer; OVCAR, ovarian cancer; PDAC, pancreatic ductal adenocarcinomas; PRCA, prostate cancer; RCC, renal cell carcinoma; SARC, sarcoma; URO, urothelial carcinoma.

1 XGBoost was used to train a supervised model based on the top 25 variables for stratifying specific cancer phenotypes.

2 Main cluster refers to the cluster that has the most percentage in each cancer phenotype in different cohorts.

**Table 3:** **Important features of supervised model for predicting different cancer phenotypes**.

Predicting Outcome	Top 3 Genes 1	Functional Annotation Of Predicting Genes (Top 3) 2
Cancer Type		
BRCA	ATP6V1A	Sulfur metabolism
	CLIC4	Other glycan degradation
	CBX7	Collecting duct acid secretion
CHOL	ARHGAP26	Fluid shear stress and atherosclerosis
	ENSG00000248538	Salivary secretion
	CNN1	Aldosterone synthesis and secretion
CRC	UTY	Pertussis
	IGF2R	Shigellosis
	CXCL8	Hematopoietic cell lineage
ENDO	RPS4Y1	IL-17 signaling pathway
	ENSG00000187653	Amoebiasis
	C1orf87	NF-kappa B signaling pathway
ESO	TNFSF4	Neurotrophin signaling pathway
	HPF1	Natural killer cell mediated cytotoxicity
	SLC3A2	mTOR signaling pathway
GLIO	DDX3Y	Fluid shear stress and atherosclerosis
	FKBP5	Sulfur metabolism
	IL1R2	Coronavirus disease - COVID-19
HCC	ENSG00000269028	Pertussis
	CTNS	Hematopoietic cell lineage
	MALAT1	Cytokine-cytokine receptor interaction
HNSSC	UTY	Systemic lupus erythematosus
	MTCO2P12	Cell adhesion molecules
	FARSA	Regulation of actin cytoskeleton
LYM	DPY19L1	NF-kappa B signaling pathway
	S100A8	Osteoclast differentiation
	NFKBIA	Apoptosis
MELA	KDM5D	Cholesterol metabolism
	PADI2	Sulfur metabolism
	UTY	Primary bile acid biosynthesis
MM	ENO2	Th1 and Th2 cell differentiation
	CD69	Th17 cell differentiation
	IL2RB	HIF-1 signaling pathway
NSCLC	ENSG00000187653	mTOR signaling pathway
	MTCO2P12	Collecting duct acid secretion
	ANKRD36C	Citrate cycle (TCA cycle)
OVCAR	EIF1AY	IL-17 signaling pathway
	MITF	NF-kappa B signaling pathway
	ENSG00000248538	Cell cycle
PDAC	C1orf87	NOD-like receptor signaling pathway
	WFDC1	Amoebiasis
	RPS4Y1	Transcriptional misregulation in cancer
PRCA	UTY	RIG-I-like receptor signaling pathway
	TSHZ2	Cytosolic DNA-sensing pathway
	TTC7A	Neurotrophin signaling pathway
RCC	SRRM2	Chagas disease
	OSBP2	Phospholipase D signaling pathway
	SIAE	Malaria
SARC	ARHGEF1	Platelet activation
	MIIP	Vascular smooth muscle contraction
	ATG16L2	alpha-Linolenic acid metabolism
URO	WDFY4	Ubiquitin mediated proteolysis
	POLG	SNARE interactions in vesicular transport
	SBF1	Base excision repair
Cancer Stage		
I Stage	TMSB4XP4	Transcriptional misregulation in cancer
	CXCL8	Bladder cancer
	HBD	Ether lipid metabolism
II Stage	BTBD11	Influenza A
	WFDC1	Coronavirus disease - COVID-19
	C1orf87	Rheumatoid arthritis
III Stage	TMEM176B	Transcriptional misregulation in cancer
	ANKRD26	Cocaine addiction
	ITK	Glutathione metabolism
IV Stage	TMSB4XP4	Coronavirus disease - COVID-19
	HSD17B3	IL-17 signaling pathway
	MTCO1P12	Relaxin signaling pathway
Early Cancer (I + II Stage)	CPNE4	Influenza A
	CXCL8	Legionellosis
	GFM1	Coronavirus disease - COVID-19

Abbreviation: KEGG, Kyoto Encyclopedia of Genes and Genomes.

1 Top 3 genes are the 3 most important features included in supervised models.

2 Functional annotations of 25 predicting genes in supervised models are based on KEGG.

#### 3.1.6 Evaluation with the confounding variables

Based on the analysis of estimated marginal means, we determined that the predicted probability for each cluster, showed no significant correlation with the library size, age, and gender (Fig. S7a-b, Fig. S8a online). With regard to institution analysis, it was worth noting that the small sample size of institution 6 (*n* ≤ 5) might affect the final prediction probabilities, while the curves of other institutions displayed a similar slope pattern, indicating that the clusters were less likely to be primarily influenced by confounding variables (Fig. S8b online).

### 3.2 Discussion

In this cross-national and multicenter study, we included the TEP transcriptomic profile of 1628 cancer individuals in the analysis, and utilized unsupervised ML to stratify them into three novel clusters. Subsequently, supervised models were employed to precisely identify clusters across the derivation, evaluation and validation cohorts. The cluster results exhibited robustness and generalizability, as demonstrated by consistent patterns of important gene expression, cancer type prevalence, and biological annotations across all three cohorts. Furthermore, supervised ML was used to identify the important mRNA features and pathways associated with different cancer types and stages. Notably, there currently exists no classification system for pan-cancer TEPs based on transcriptomic profiles. The results proved that data-driven unsupervised and supervised ML could help us to better understand the variability of the education mechanism of pan-cancer. By utilizing the novel classification of cluster phenotypes, we can further investigate the biological characteristics of each cluster and explore their potential clinical applications, such as cancer diagnosis, prognosis, and treatment guidance.

The physical and functional interactions between cancer and platelets result in biomolecular changes in platelets, thereby enabling them to play critical biological roles in tumor angiogenesis, growth, and metastasis [6,16]. Additionally, cancer is frequently associated with an elevated risk of both thrombosis and bleeding, with epidemiological studies reporting an increased incidence of venous thromboembolism in affected individuals. Despite extensive research efforts, the underlying mechanisms driving these observations remain largely unclear. It's emerging to reveal the potential molecular pattern and the corresponding biology features of education results from different type of cancers.

Accumulating studies indicate that the platelet transcriptome contains a rich repertoire of biomarkers that clarify many aspects relevant to cancer [17,18]. In addition to early screening and diagnoses, the platelet transcriptome could also be used to identify different types of cancers [5,18], metastasis [5,18], track disease severity [19], predict marrow fibrosis in myeloproliferative neoplasms [20], predict CATs [21] and distinguish cancers with mutant KRAS and EGFR [18]. The changes and the variability of platelet transcriptome can be associated with heterogeneity of cancers and can serve as biomarkers for estimating different education mechanisms. The major contribution of our work was to build novel classification system for TEP to investigate the biology variability based on the platelet transcriptome in a large pan-cancer cohort. The results exhibited high generality and could be further applied to different types of cancers. Further observational studies can investigate the association between these clusters and cancer progression, prognosis, CATs, treatment resistance (both chemotherapy and immunotherapy), and immune cell infiltration. In the future, based on the cluster system, we may obtain more information about cancer without truly contacting it (like puncture biopsy or transurethral bladder tumor resection).

Finally, we determined *N* = 3 as the robust cluster number based on the CH and DB scores. The CH score measures the quality of separation between clusters, while the DB score quantifies the scatter within each cluster [8]. A higher CH value or lower DB value indicates a better and more robust clustering. Cluster 1 (*n* = 346), mainly participated in drug metabolism cytochrome P450, metabolism of xenobiotics by cytochrome P450 and glutathione metabolism. The core enrichment genes of the first two pathways were GSTM5, GSTA1, GSTO1, GSTM4, MGST3 and MAOB, which were all up-regulated in Cluster 1 compared with other clusters (Table S6 online). GSTM5, GSTA1, GSTO1, GSTM4 and MGST3 are also involved in glutathione metabolism pathway, which is proved to be associated with inactivation of platinum compounds [22] (Table S6 online). The cytosolic glutathione S-transferase (GST) family primarily participates in the detoxification of reactive electrophiles, cellular signaling, anti-apoptotic activity, and anti-inflammatory responses [23]. Additionally, this family of enzymes has been implicated in cancer development and cisplatin-resistance [24]. Previous study found that reduced expression of GSTA1 were associated with increased risk of HCC and oral cancer [25,26]. Overexpression of GSTM4 was found to be associated with lower survival rates in lung cancer treated with platinum-containing agents and decreased cisplatin sensitivity [22]. However, GSTM5 has been demonstrated to have anti-cancer effects in bladder cancer cells and lung adenocarcinoma [27,28]. Low expression levels of GSTM5 have been associated with significantly shorter overall survival in lung adenocarcinoma [28]. Further investigation is needed to determine the exact impact of Cluster 1 on cancer. Interestingly, in this study, we observed a notably low percentage of Cluster 1 in urologic cancers (0% in URO, 14.3% in RCC, 0% in PRCA). In future research, we will explore the relationship between each cluster and the sensitivity of neoadjuvant chemotherapy or immunotherapy in urologic cancers.

Cluster 2 (*n* = 538 in total) mainly participated in ribosome, spliceosome, and primary immunodeficiency. Recent studies have provided mounting evidence that cancer cells possess a distinct class of ribosomes known as onco-ribosomes, which facilitate oncogenic translation programs, modulate cellular functions, and promote metabolic rewiring [29]. Aberrant ribosomes could be associated with poor prognosis and treatment failure in cancer [30]. Our findings showed that several ribosomal protein genes, including RPL9, RPL11 and RPL6, among others, were up-regulated in Cluster 2 (Table S6 online). Even though platelets lack nuclei to produce new mRNA and ribosomes, protein synthesis can continue through dynamic regulation of a ribosome rescue pathway, which may be one of the education mechanisms [31]. Similar to ribosome-related genes, spliceosome-related genes such as SNRPA, SNRPF, NRP1 and DDX5, among others, were found to be up-regulated in Cluster 2 (Table S6 online). Splicing is the major regulatory mechanism for TEP mRNA expression [32]. When activated by the cancer microenvironment, the transcripts can be specifically spliced into mature mRNA and translated into thousands of different proteins in platelets [1,32]. The alternation of platelet spliceosome can be biomarker for diagnosis of cancer [4]. However, there have been limited studies investigating the effects of this phenomenon on cancer. As for primary immunodeficiency, CD3D, IL2RG, IL7R, CD19, and CD4 were up-regulated in Cluster 2 (Table S6 online). Tumor-infiltrating lymphocytes represent a vital component of the tumor microenvironment and are regarded as an important prognostic factor in cancer. CD3D, which plays vital functions in T cell antigen recognition and signal transduction, has been proved to be associated with immune checkpoints and predict favorable clinical outcome in colon cancer and HNSSC [33,34]. Along with CD4, CD3D also can predict immune infiltration and the best prognosis in gastric cancer [35]. From this perspective, Cluster 2 may be a favorable candidate for immunotherapy. However, further investigation is needed to explore the potential association between Cluster 2 and tumor-infiltrating lymphocytes in cancer, as well as the treatment response.

Cluster 3 (*n* = 744 in total) mainly participated in gap junction and focal adhesion. Loss of gap junction communication is a common phenomenon observed in cancers [36]. Meanwhile, gap junctions among endothelial cells, platelets, and leukocytes also play a pivotal role in blood coagulation [37]. TUBB, TUBB1 were found to be up-regulated in Cluster 3 (Table S6 online). It has been indicated that TUBB mediates cell cycle, epithelial-mesenchymal transition (EMT), proliferation, metastasis, and invasion in MELA [38]. In individuals with BRCA that are resistant to chemotherapy, TUBB is also upregulated [38,39]. TUBB1 is the predominant isoform of tubulin expressed in megakaryocytes and platelets, which is important for proplatelet formation and maintenance of platelet shape. TUBB1 is also proved to be correlated with tumor infiltrating immune cells and can predict prognosis in osteosarcoma [40]. Regarding the second pathway, focal adhesion is an essential step in cancer cell migration and invasion, and can promote tumorigenesis and metastasis via phosphorylation and protein-protein interactions. In the core enrichment genes of focal adhesion, we found that MAPK1, PTEN, ITGB1 and PIK3CG were up-regulated. MAPK1 is proved to be a potential target for inhibiting the progression and metastasis of gastric cancer and BRCA [41,42]. ITGB1 is associated with proliferation and invasion of gastric cancer, and is proved to enhance radioresistance of NSCLC by modulating EMT. The association between Cluster 3 and cancer EMT, metastasis and formation of CATs should be further studied.

This study had several strengths. First, this study was the first to build a stratification system for TEP based on platelet transcriptome profiles, which could help us to better investigate the variability of the education mechanism of pan-cancer. To our knowledge, no similar study has been conducted before, except for studies on platelet variability in myeloproliferative neoplasms [7]. However, its variability was based more on classical myeloproliferative neoplasm subtypes, including essential thrombocythemia, polycythemia vera, and primary myelofibrosis. Transcriptome profiles, which provided up to 5000 features, and data-driven patterns could help us better classify and find similar education mechanisms for pan-cancer. Second, thanks to the contribution of the original study by In't Veld et al. [5], we were able to involve the TEP data of 1628 patients across the United States and Europe. Adequate data were obtained for ML, which ensured the stability of the model and prevented bias in small samples. Based on their study, we validated the accuracy of the TEP transcriptome profiles in the diagnosis of each cancer phenotype. Third, the GMM allows explicit analysis of mRNA profiles and subgroups of patients based on similarity in expression patterns among multiple genes. It provides a “first aid” solution for resolving TEP variability and can be applied to huge amounts of transcriptomic data because of its flexibility [43].

This study also had several limitations. First, the number of different types of cancer varied significantly. The most involved cancers were NSCLC (*n* = 522), whereas ESO (*n* = 15), HCC (*n* = 23), and LYM (*n* = 20) accounted for only a small proportion, resulting in an imbalance. However, the number of cancer types may reflect advances in TEP research. NSCLC, GLIO, and BRCA have been extensively studied, but few studies have focused on the potential education mechanisms of other types of cancers. Further studies are needed to investigate the association between TEP and other cancer types. Second, only one type of hematological malignancy was included in this study. Compared with other solid tumor diseases, hematological malignancies can have a greater effect on platelets and megakaryocytes. Whether TEP play a role in hemostasis, thrombosis, and the progression of cancer should be further studied. Third, the data involved in this study only included diagnostic outcomes and lacked prognostic outcomes. We are interested in investigating whether TEP or the cluster could serve as a prognostic or therapeutic indicator of cancer, which should be conducted in further prospective studies.

## 4 Conclusion

In this study, we aimed to build the first pan-cancer TEP stratification system based on data-driven patterns obtained from ML and platelet transcriptome profiles. These clusters could help us better understand the variability of the pan-cancer education mechanism. Further studies should focus on investigating the association between the identified clusters and cancer progression and CATs.

**Table:** Abbreviations

TEP	tumor-educated platelets
CAT	cancer-associated thrombosis
ML	machine learning
BRCA	breast cancer
CHOL	cholangiocarcinoma
CRC	colorectal cancer
ENDO	endometrial cancer
ESO	esophageal cancer
GLIO	glioma
HCC	hepatocellular carcinoma
HNSSC	head and neck squamous cell carcinoma
LYM	lymphoma
MELA	melanoma
MM	multiple myeloma
NSCLC	non-small cell lung cancer
OVCAR	ovarian cancer
PDAC	pancreatic ductal adenocarcinoma
PRCA	prostate cancer
RCC	renal cell carcinoma
SARC	sarcoma
URO	urothelial carcinoma
XGBoost	extreme gradient boosting
GMM	gaussian mixture model
CH	Calinski–Harabasz
DB	Davies–Bouldin
AUC	area under the receiver operating characteristic curve
SMOTE	synthetic minority oversampling technique
ROC	receiver operating characteristic curves
GSEA	gene set enrichment analysis
KEGG	Kyoto Encyclopedia of Genes and Genomes
FDR	false discovery rate
IQR	interquartile ranges
DEG	differentially expressed genes
OR	odds ratio
CI	confidence interval.

## Role of the funding source

The funders of this research had no role in the study design, management, provision of study materials, data collection, data analysis, data interpretation, manuscript writing, preparation, review, or approval of this manuscript, and decision to submit the manuscript for publication.

## Declaration of competing interest

The authors declare that they have no conflicts of interest in this work.

## Appendix Supplementary materials

Image, application 1

Image, application 2
