# Supplementary material for: Unsupervised and supervised machine learning to identify variability of tumor-educated platelets and association with pan-cancer: A cross-national study
Source: Fundam Res. 2023 Nov 2;6(1):498–508. doi: 10.1016/j.fmre.2023.09.004 (PMC12869750; doi:10.1016/j.fmre.2023.09.004)
Supplement: Supplementary file 1 [file mmc1.docx]

**Unsupervised and Supervised Machine Learning to Identify Variability of Tumor-Educated Platelets and Association with Pan-Cancer: A Cross-National Study**

Supplementary Files

[Supplementary Table 1. Baseline characteristic of patients stratified by clusters in derivation cohort. 3](#_Toc144941716)

[Supplementary Table 2. Baseline characteristic of patients stratified by clusters in evaluation cohort. 5](#_Toc144941717)

[Supplementary Table 3. Baseline characteristic of patients stratified by clusters in validation cohort. 7](#_Toc144941718)

[Supplementary Table 4. Cancer classification score in different clusters in total cohorts 9](#_Toc144941719)

[Supplementary Table 5. Association between clusters and cancer phenotypes based on logistic regression. 10](#_Toc144941720)

[Supplementary Table 6. Core enrichment genes of Top 3 KEGG pathway in each cluster in derivation cohort. 12](#_Toc144941721)

[Supplementary Figure 1. Calinski Harabasz Score and Davies Bouldin Score in gaussian mixture model. 13](#_Toc144941722)

[Supplementary Figure 2. Bar chart of clusters in each cancer type in derivation, evaluation, and validation cohort. 14](#_Toc144941723)

[Supplementary Figure 3. Bar chart of clusters in each cancer stage in derivation, evaluation, and validation cohort. 15](#_Toc144941724)

[Supplementary Figure 4. Violin plots of cancer classification score in different clusters in total cohort 16](#_Toc144941725)

[Supplementary Figure 5. Gene set enrichment analysis of each cluster in evaluation cohort based on KEGG. 17](#_Toc144941726)

[Supplementary Figure 6. Heatmap of expression of genes that participated in platelet activation and complement and coagulation cascades in three clusters. 18](#_Toc144941727)

[Supplementary Figure 7. Evaluation of potential effect of library size and age on prediction probabilities for 3 clusters. 19](#_Toc144941728)

[Supplementary Figure 8. Evaluation of potential effect of sex and institution on prediction probabilities for 3 clusters. 20](#_Toc144941730)

# Supplementary Table 1. Baseline characteristic of patients stratified by clusters in derivation cohort.

| **Items** | **Cluster 1**  **(n = 136)** | **Cluster 2**  **(n = 217)** | **Cluster 3**  **(n = 299)** | ***P*-value** |
| --- | --- | --- | --- | --- |
| Sex (Male) | 68 (50.0%) | 124 (57.4%) | 135 (45.3%) | **0.025** |
| Age | 62.50 [55.75, 68.00] | 65.00 [57.00, 71.00] | 61.00 [52.00, 69.00] | **0.004** |
| Disease Status |  |  |  |  |
| BRCA | 5 (3.7%) | 0 (0.0%) | 39 (13.0%) | **<0.001** |
| CHOL | 10 (7.4%) | 3 (1.4%) | 19 (6.4%) | **0.012** |
| CRC | 8 (5.9%) | 7 (3.2%) | 20 (6.7%) | 0.217 |
| ENDO | 2 (1.5%) | 6 (2.8%) | 10 (3.3%) | 0.543 |
| ESO | 0 (0.0%) | 3 (1.4%) | 2 (0.7%) | 0.338 |
| GLIO | 16 (11.8%) | 14 (6.5%) | 24 (8.0%) | 0.207 |
| HNSSC | 6 (4.4%) | 23 (10.6%) | 5 (1.7%) | **<0.001** |
| HCC | 0 (0.0%) | 0 (0.0%) | 10 (3.3%) | **0.002** |
| LYM | 2 (1.5%) | 0 (0.0%) | 6 (2.0%) | 0.119 |
| MELA | 4 (2.9%) | 6 (2.8%) | 13 (4.3%) | 0.577 |
| MM | 0 (0.0%) | 8 (3.7%) | 4 (1.3%) | **0.029** |
| NSCLC | 50 (36.8%) | 94 (43.3%) | 63 (21.1%) | **<0.001** |
| OVCAR | 11 (8.1%) | 7 (3.2%) | 41 (13.7%) | **<0.001** |
| PDAC | 19 (14.0%) | 15 (6.9%) | 22 (7.4%) | **0.041** |
| PRCA | 0 (0.0%) | 6 (2.8%) | 4 (1.3%) | 0.112 |
| RCC | 3 (2.2%) | 0 (0.0%) | 11 (3.7%) | **0.017** |
| SARC | 0 (0.0%) | 16 (7.4%) | 1 (0.3%) | **<0.001** |
| URO | 0 (0.0%) | 9 (4.1%) | 5 (1.7%) | **0.024** |
| Cancer Stage |  |  |  |  |
| I Stage | 6 (5.1%) | 15 (8.3%) | 27 (10.9%) | 0.182 |
| II Stage | 22 (18.8%) | 17 (9.4%) | 36 (14.6%) | 0.065 |
| III Stage | 19 (16.2%) | 30 (16.7%) | 43 (17.4%) | 0.957 |
| IV Stage | 70 (59.8%) | 118 (65.6%) | 141 (57.1%) | 0.207 |

Age is represented by median with interquartile range (IQR) and compared with Kruskal-Wallis test. Categorical variables are expressed as percentages, compared with chi-square tests. A two-sided *P* < 0.05 was considered statistically significant.

Abbreviation: BRCA, breast cancer; CHOL, cholangiocarcinoma; CRC, colorectal cancer; ENDO, endometrial cancer; ESO, esophageal cancer; GLIO, glioma; HCC, hepatocellular carcinoma; HNSSC, head and neck squamous cell carcinoma; LYM, lymphoma; MELA, melanoma; MM, multiple myeloma; NSCLC, non-small cell lung cancer; OVCAR, ovarian cancer; PDAC, pancreatic ductal adenocarcinomas; PRCA, prostate cancer; RCC, renal cell carcinoma; SARC, sarcoma; URO, urothelial carcinoma.

# Supplementary Table 2. Baseline characteristic of patients stratified by clusters in evaluation cohort.

| **Items** | **Cluster 1**  **(n = 76)** | **Cluster 2**  **(n = 113)** | **Cluster 3**  **(n = 136)** | ***P*-value** |
| --- | --- | --- | --- | --- |
| Sex (Male) | 41 (53.9%) | 61 (54.5%) | 54 (39.7%) | **0.035** |
| Age | 65.00 [60.00, 71.00] | 64.00 [56.00, 70.00] | 64.50 [55.75, 73.25] | 0.429 |
| Disease Status |  |  |  |  |
| BRCA | 2 (2.6%) | 0 (0.0%) | 15 (11.0%) | **<0.001** |
| CHOL | 5 (6.6%) | 4 (3.5%) | 10 (7.4%) | 0.422 |
| CRC | 8 (10.5%) | 2 (1.8%) | 10 (7.4%) | **0.037** |
| ENDO | 1 (1.3%) | 4 (3.5%) | 3 (2.2%) | 0.607 |
| ESO | 0 (0.0%) | 2 (1.8%) | 0 (0.0%) | 0.151 |
| GLIO | 9 (11.8%) | 8 (7.1%) | 7 (5.1%) | 0.200 |
| HNSSC | 1 (1.3%) | 19 (16.8%) | 9 (6.6%) | **0.001** |
| HCC | 1 (1.3%) | 0 (0.0%) | 2 (1.5%) | 0.443 |
| LYM | 1 (1.3%) | 1 (0.9%) | 2 (1.5%) | 0.914 |
| MELA | 2 (2.6%) | 3 (2.7%) | 11 (8.1%) | 0.082 |
| MM | 0 (0.0%) | 4 (3.5%) | 1 (0.7%) | 0.093 |
| NSCLC | 32 (42.1%) | 41 (36.3%) | 27 (19.9%) | **0.001** |
| OVCAR | 2 (2.6%) | 2 (1.8%) | 19 (14.0%) | **<0.001** |
| PDAC | 9 (11.8%) | 2 (1.8%) | 13 (9.6%) | **0.015** |
| PRCA | 0 (0.0%) | 4 (3.5%) | 4 (2.9%) | 0.273 |
| RCC | 1 (1.3%) | 0 (0.0%) | 2 (1.5%) | 0.443 |
| SARC | 2 (2.6%) | 11 (9.7%) | 1 (0.7%) | **0.002** |
| URO | 0 (0.0%) | 6 (5.3%) | 0 (0.0%) | **0.003** |
| Cancer Stage |  |  |  |  |
| I Stage | 2 (3.0%) | 7 (7.4%) | 15 (12.4%) | 0.080 |
| II Stage | 7 (10.6%) | 8 (8.4%) | 13 (10.7%) | 0.833 |
| III Stage | 11 (16.7%) | 17 (17.9%) | 21 (17.4%) | 0.980 |
| IV Stage | 46 (69.7%) | 63 (66.3%) | 72 (59.5%) | 0.331 |

Age is represented by median with interquartile range (IQR) and compared with Kruskal-Wallis test. Categorical variables are expressed as percentages, compared with chi-square tests. A two-sided *P* < 0.05 was considered statistically significant.

Abbreviation: BRCA, breast cancer; CHOL, cholangiocarcinoma; CRC, colorectal cancer; ENDO, endometrial cancer; ESO, esophageal cancer; GLIO, glioma; HCC, hepatocellular carcinoma; HNSSC, head and neck squamous cell carcinoma; LYM, lymphoma; MELA, melanoma; MM, multiple myeloma; NSCLC, non-small cell lung cancer; OVCAR, ovarian cancer; PDAC, pancreatic ductal adenocarcinomas; PRCA, prostate cancer; RCC, renal cell carcinoma; SARC, sarcoma; URO, urothelial carcinoma.

# Supplementary Table 3. Baseline characteristic of patients stratified by clusters in validation cohort.

| **Items** | **Cluster 1**  **(n = 134)** | **Cluster 2**  **(n = 208)** | **Cluster 3**  **(n = 309)** | ***P*-value** |
| --- | --- | --- | --- | --- |
| Sex (Male) | 66 (49.6%) | 119 (57.2%) | 138 (44.7%) | **0.020** |
| Age | 64.00 [56.00, 70.00] | 63.00 [56.00, 70.00] | 62.00 [53.00, 69.00] | 0.340 |
| Disease Status |  |  |  |  |
| BRCA | 4 (3.0%) | 1 (0.5%) | 27 (8.7%) | **<0.001** |
| CHOL | 14 (10.4%) | 2 (1.0%) | 18 (5.8%) | **<0.001** |
| CRC | 6 (4.5%) | 0 (0.0%) | 24 (7.8%) | **<0.001** |
| ENDO | 2 (1.5%) | 5 (2.4%) | 6 (1.9%) | 0.837 |
| ESO | 1 (0.7%) | 7 (3.4%) | 0 (0.0%) | **0.003** |
| GLIO | 15 (11.2%) | 13 (6.2%) | 26 (8.4%) | 0.269 |
| HNSSC | 1 (0.7%) | 27 (13.0%) | 10 (3.2%) | **<0.001** |
| HCC | 1 (0.7%) | 0 (0.0%) | 9 (2.9%) | **0.022** |
| LYM | 0 (0.0%) | 1 (0.5%) | 7 (2.3%) | 0.069 |
| MELA | 3 (2.2%) | 5 (2.4%) | 21 (6.8%) | **0.023** |
| MM | 1 (0.7%) | 5 (2.4%) | 8 (2.6%) | 0.449 |
| NSCLC | 57 (42.5%) | 84 (40.4%) | 74 (23.9%) | **<0.001** |
| OVCAR | 14 (10.4%) | 16 (7.7%) | 32 (10.4%) | 0.551 |
| PDAC | 14 (10.4%) | 10 (4.8%) | 22 (7.1%) | 0.139 |
| PRCA | 0 (0.0%) | 8 (3.8%) | 9 (2.9%) | 0.084 |
| RCC | 0 (0.0%) | 1 (0.5%) | 10 (3.2%) | **0.014** |
| SARC | 1 (0.7%) | 16 (7.7%) | 5 (1.6%) | **<0.001** |
| URO | 0 (0.0%) | 7 (3.4%) | 1 (0.3%) | **0.003** |
| Cancer Stage |  |  |  |  |
| I Stage | 7 (6.2%) | 15 (8.3%) | 17 (6.7%) | 0.756 |
| II Stage | 18 (16.1%) | 16 (8.8%) | 29 (11.5%) | 0.170 |
| III Stage | 16 (14.3%) | 37 (20.4%) | 41 (16.2%) | 0.337 |
| IV Stage | 71 (63.4%) | 113 (62.4%) | 166 (65.6%) | 0.781 |

Age is represented by median with interquartile range (IQR) and compared with Kruskal-Wallis test. Categorical variables are expressed as percentages, compared with chi-square tests. A two-sided *P* < 0.05 was considered statistically significant.

Abbreviation: BRCA, breast cancer; CHOL, cholangiocarcinoma; CRC, colorectal cancer; ENDO, endometrial cancer; ESO, esophageal cancer; GLIO, glioma; HCC, hepatocellular carcinoma; HNSSC, head and neck squamous cell carcinoma; LYM, lymphoma; MELA, melanoma; MM, multiple myeloma; NSCLC, non-small cell lung cancer; OVCAR, ovarian cancer; PDAC, pancreatic ductal adenocarcinomas; PRCA, prostate cancer; RCC, renal cell carcinoma; SARC, sarcoma; URO, urothelial carcinoma.

# Supplementary Table 4. Cancer classification score in different clusters in total cohorts

|  | cluster 1 | cluster 2 | cluster 3 | *P*-value |
| --- | --- | --- | --- | --- |
|  | (n = 346) | (n = 538) | (n = 744) |  |
| Cancer classification Score (median [IQR]) | 0.96 [0.87, 0.99] | 0.95 [0.86, 0.98] | 0.91 [0.66, 0.98] | <0.001 |

Cancer classification score is represented by a median with interquartile range (IQR) and compared with the Kruskal-Wallis test. The results show that cancer classification score in cluster 3 is lower than that in cluster 1 and 2.

# Supplementary Table 5. Association between clusters and cancer phenotypes based on logistic regression.

| **Predicting Outcome** | **Cohort** | **Adjusted ^1^** | **Significant Cluster** | **OR (95% CI)** | ***P*-value** |
| --- | --- | --- | --- | --- | --- |
| Cancer Type |  |  |  |  |  |
| BRCA | Training | Non-adjusted | cluster 3 | 3.93 (95% CI = 1.65-11.61) | **0.005** |
|  | Training | Adjusted | cluster 3 | 4.06 (95% CI = 1.65-12.24) | **0.005** |
|  | Evaluation | Non-adjusted | cluster 3 | 4.59 (95% CI = 1.25-29.63) | **0.047** |
|  | Validation | Non-adjusted | cluster 3 | 3.11 (95% CI = 1.19-10.69) | **0.038** |
| CHOL | Training | Non-adjusted | cluster 2 | 0.18 (95% CI = 0.04-0.59) | **0.009** |
|  | Training | Adjusted | cluster 2 | 0.16 (95% CI = 0.04-0.55) | **0.007** |
|  | Validation | Non-adjusted | cluster 2 | 0.08 (95% CI = 0.01-0.30) | **0.001** |
|  | Validation | Adjusted | cluster 2 | 0.08 (95% CI = 0.01-0.31) | **0.001** |
| CRC | Evaluation | Non-adjusted | cluster 2 | 0.15 (95% CI = 0.02-0.63) | **0.020** |
|  | Evaluation | Adjusted | cluster 2 | 0.16 (95% CI = 0.02-0.67) | **0.023** |
| GLIO | Training | Adjusted | cluster 3 | 0.44 (95% CI = 0.21-0.93) | **0.030** |
| HNSSC | Training | Non-adjusted | cluster 2 | 2.57 (95% CI = 1.08-7.11) | **0.046** |
|  | Evaluation | Non-adjusted | cluster 2 | 15.16 (95% CI = 3.04-275.49) | **0.009** |
|  | Evaluation | Adjusted | cluster 2 | 16.26 (95% CI = 3.23-296.45) | **0.007** |
|  | Validation | Non-adjusted | cluster 2 | 19.84 (95% CI = 4.15-356.14) | **0.004** |
|  | Validation | Adjusted | cluster 2 | 18.63 (95% CI = 3.88-334.84) | **0.004** |
| NSCLC | Training | Non-adjusted | cluster 3 | 0.46 (95% CI = 0.29-0.72) | **0.001** |
|  | Training | Adjusted | cluster 3 | 0.47 (95% CI = 0.30-0.73) | **0.001** |
|  | Evaluation | Non-adjusted | cluster 3 | 0.34 (95% CI = 0.18-0.63) | **0.001** |
|  | Evaluation | Adjusted | cluster 3 | 0.33 (95% CI = 0.18-0.62) | **0.001** |
|  | Validation | Non-adjusted | cluster 3 | 0.43 (95% CI = 0.28-0.66) | **<0.001** |
|  | Validation | Adjusted | cluster 3 | 0.44 (95% CI = 0.28-0.67) | **<0.001** |
| OVCAR | Evaluation | Non-adjusted | cluster 3 | 6.01 (95% CI = 1.68-38.41) | **0.018** |
|  | Evaluation | Adjusted | cluster 3 | 4.95 (95% CI = 1.32-32.35) | **0.039** |
| PDAC | Training | Non-adjusted | cluster 2 | 0.46 (95% CI = 0.22-0.93) | **0.032** |
|  | Training | Non-adjusted | cluster 3 | 0.49 (95% CI = 0.26-0.95) | **0.031** |
|  | Training | Adjusted | cluster 2 | 0.41 (95% CI = 0.20-0.85) | **0.017** |
|  | Evaluation | Non-adjusted | cluster 2 | 0.13 (95% CI = 0.02-0.54) | **0.012** |
|  | Evaluation | Adjusted | cluster 2 | 0.14 (95% CI = 0.02-0.58) | **0.015** |
| SARC | Validation | Non-adjusted | cluster 2 | 11.08 (95% CI = 2.22-201.25) | **0.020** |
|  | Validation | Adjusted | cluster 2 | 10.99 (95% CI = 2.19-199.76) | **0.021** |
| Cancer Stage |  |  |  |  |  |
| I Stage | Evaluation | Non-adjusted | cluster 3 | 4.53 (95% CI = 1.22-29.33) | **0.050** |
| II Stage | Training | Non-adjusted | cluster 2 | 0.45 (95% CI = 0.23-0.89) | **0.022** |
|  | Training | Adjusted | cluster 2 | 0.45 (95% CI = 0.23-0.89) | **0.022** |

^1^ Models were adjusted for age and gender.

Abbreviation: OR, odds ratio; CI, confidence interval; BRCA, breast cancer; CHOL, cholangiocarcinoma; CRC, colorectal cancer; ENDO, endometrial cancer; ESO, esophageal cancer; GLIO, glioma; HCC, hepatocellular carcinoma; HNSSC, head and neck squamous cell carcinoma; LYM, lymphoma; MELA, melanoma; MM, multiple myeloma; NSCLC, non-small cell lung cancer; OVCAR, ovarian cancer; PDAC, pancreatic ductal adenocarcinomas; PRCA, prostate cancer; RCC, renal cell carcinoma; SARC, sarcoma; URO, urothelial carcinoma.

# Supplementary Table 6. Core enrichment genes of Top 3 KEGG pathway in each cluster in derivation cohort.

| **Cluster** | **Pathway** | **Core Enrichment Genes** |
| --- | --- | --- |
| 1 | Drug Metabolism Cytochrome P450 | GSTM5, GSTA1, GSTO1, GSTM4, MGST3, MAOB |
| 1 | Metabolism of Xenobiotics by Cytochrome P450 | GSTM5, GSTA1, GSTO1, GSTM4, MGST3 |
| 1 | Glutathione Metabolism | GPX1, GPX4, GSTM5, GSTA1, GSTO1, G6PD, ODC1, GSTM4, MGST3 |
| 2 | Ribosome | RPL9, RPS21, RPS24, RPLP2, RPL18, RPL11, RPSA, RPL10A, RPL23, RPS6, RPL6, RPL37, RPL37A, RPS16, RPL5, RPL34, RPS8, RPL27, RPL7A, RPL36, RPL28, RPL24, RPL35, RPS13, RPS29, RPL32, RPL38, RPL3, RPS7, RPS4X, RPL13, RPL23A, RPS9, RPS4Y1, RPL18A, RPS2, RPL8, RPL12, RPS18, RPL19, RPS26, RPS28, RPS12, RPLP0, RPL31, RPS3A, RPS5, RPL7, RPL13A, RPS3, RPS15, RPL27A, RPS19, RPL14, RPS27A, RPS25, RPL21, RPS27, RPS15A, RPS20, RPLP1, RPL10, FAU, RSL24D1, RPS11, RPL29 |
| 2 | Spliceosome | SNRNP70, RBM25, PRPF3, U2SURP, SNRNP200, DHX16, ACIN1, SART1, PRPF38B, SNRPA1, SRSF5, TCERG1, DDX46, SRSF7, U2AF1, THOC1, CDC40, SNRPA, SNRPF, PRPF31, SNRPD1, SF3A3, PRPF6, CCDC12, SNRPD2, HNRNPA1, SF3B1, CWC15, ALYREF, SF3B3, SF3B2, PQBP1, PLRG1, LSM8, SNRPG, DDX42, DDX23, HSPA8, EIF4A3, DDX5, PRPF40A, DHX15, PUF60, HNRNPM, DHX8, CRNKL1, AQR, RBMX, PRPF38A, LSM6, SNRPB2, BCAS2, HNRNPA3, DHX38 |
| 2 | Primary Immunodeficiency | CD3D, PTPRC, IL2RG, CD3E, ZAP70, IL7R, CD19, LCK, CD79A, CD4, CIITA, DCLRE1C, ADA, RFXANK |
| 3 | Gap junction | TUBB1, ADCY3, GNAQ, EGF, PDGFC, PRKG1, PRKCB, GUCY1B1, TUBB, TUBA4A, PRKACB, SRC, MAPK1, TUBB4B, NRAS, TUBA1C, TUBA1B, MAP3K2, GNAS, RAF1, GUCY1A1, PLCB4 |
| 3 | Focal adhesion | VCL, PTK2, CAV2, EGF, PDGFC, CRKL, ACTN1, CCND3, PRKCB, CAPN2, ITGB5, PIK3CB, PIP5K1C, SRC, ITGB1, MAPK1, RAP1B, MYLK, ACTB, PIK3CG, MAPK10, ITGA2, RHOA, PPP1CB, ITGA2B, ZYX, IGF1R, MYL12A, RAF1, MYL9, FLNA, LAMB2, VWF, ACTG1, FYN, PARVB, LAMC1, ACTN4, SHC1, PIK3CA, THBS1, AKT1, BAD, MAP2K1, DIAPH1, TLN1, PPP1CC, GRB2, PIK3R1, RAP1A, ROCK2, PTEN, VAV3, ITGA6, GSK3B, CDC42, PDGFA |
| 3 | Melanoma | CDKN1A, EGF, PDGFC, E2F1, PIK3CB, MITF, MAPK1, NRAS, PIK3CG, E2F3, IGF1R, RAF1, PIK3CA, AKT1, BAD, MAP2K1, ARAF, PIK3R1, PTEN, PDGFA, RB1 |

Gene set enrichment analysis (GSEA) was performed to further explore the biological characteristics in each cluster. Gene sets with normalized enrichment score >1, P <0.05, and false discovery rate (FDR) q <0.25 were considered enriched.

# Supplementary Figure 1. Calinski Harabasz Score and Davies Bouldin Score in gaussian mixture model.


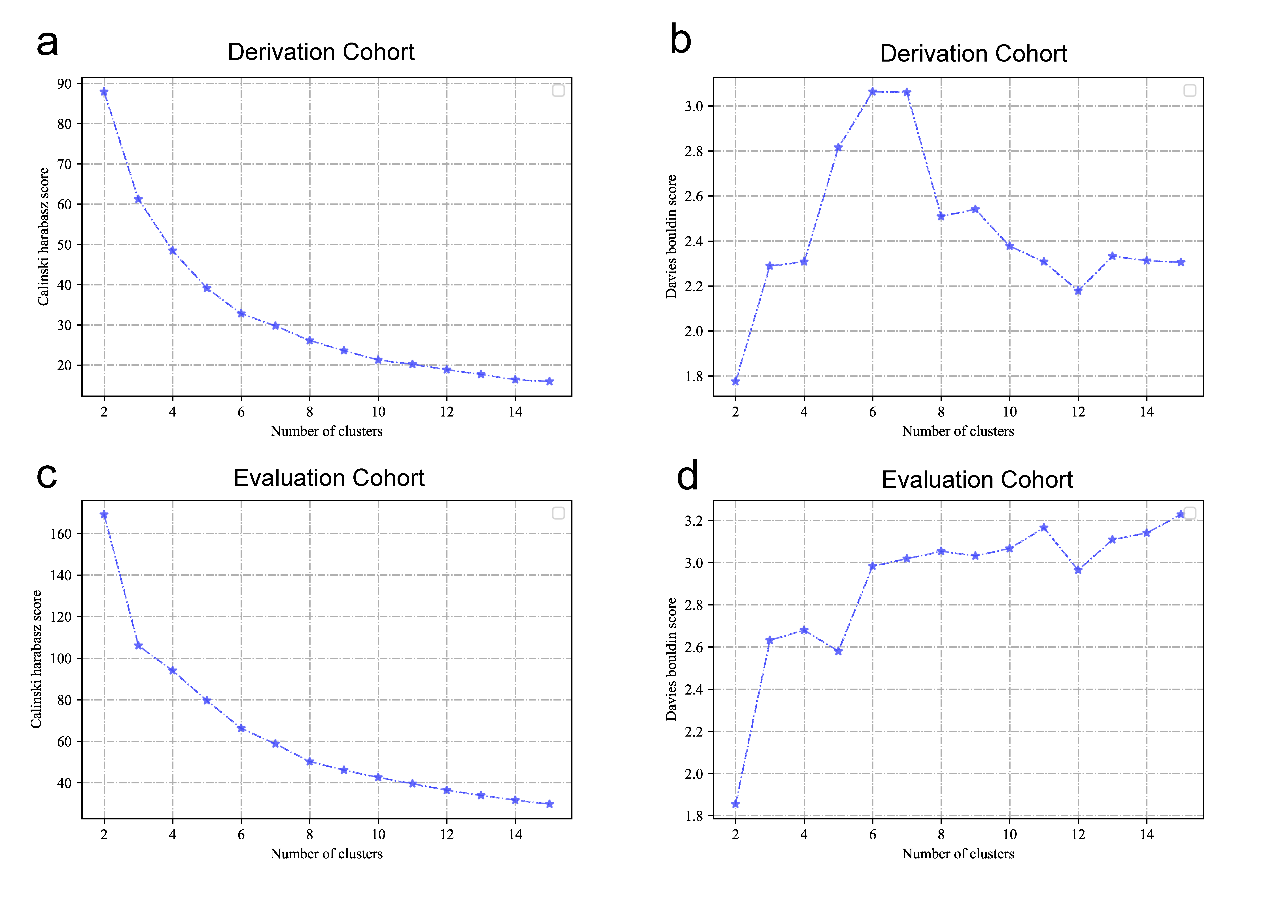


(a-b) gaussian mixture model was developed using the derivation cohort; (c-d) gaussian mixture model was developed using the evaluation cohort.

Finally, we chose n = 3 as robust cluster number based on CH and DB score. CH score is a measure of the quality of the separation between clusters while DB score measures the with-in cluster scatter. Higher CH value or lower DB value means better and robust clustering.

#
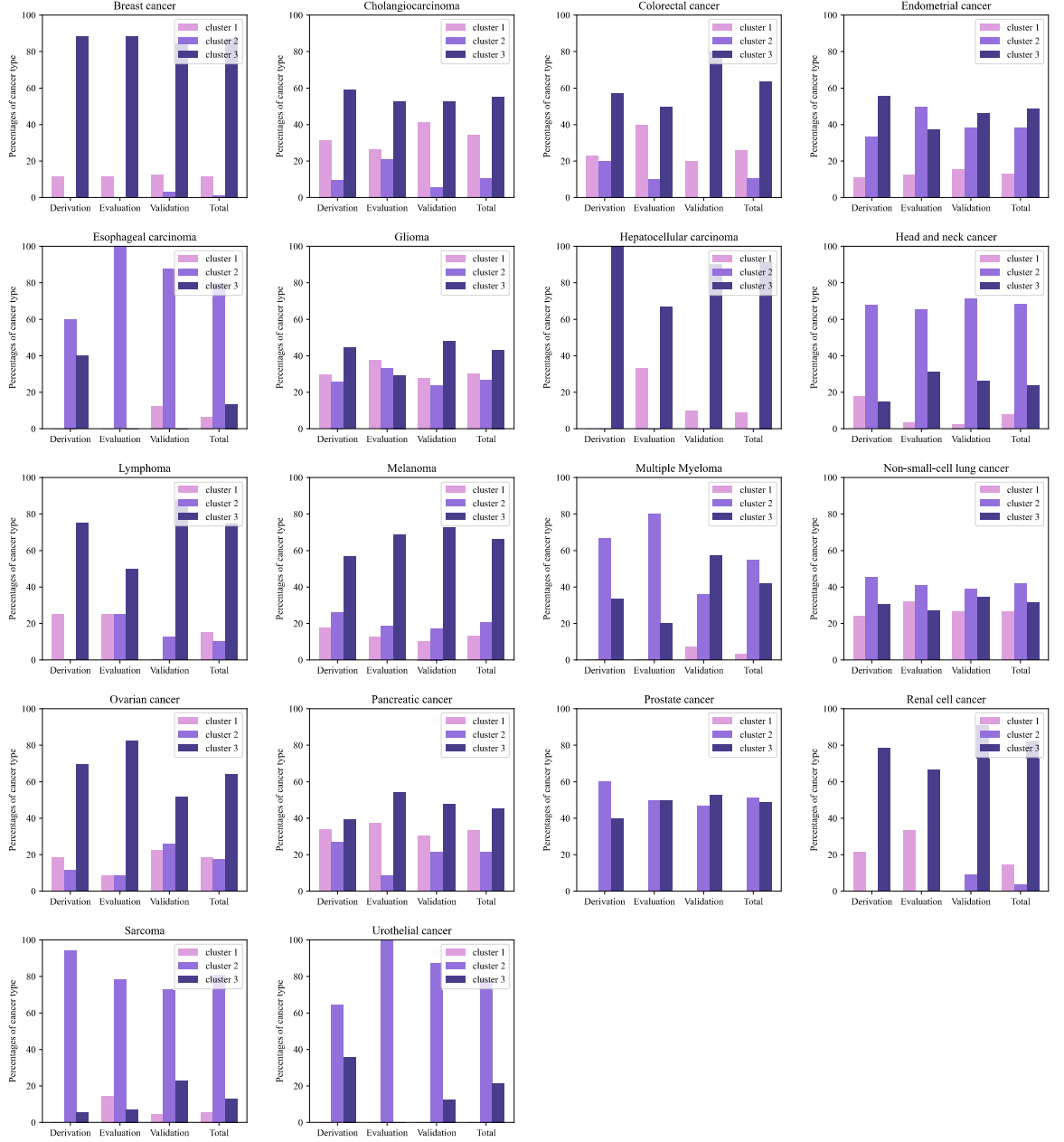
Supplementary Figure 2. Bar chart of clusters in each cancer type in derivation, evaluation, and validation cohort.

# Supplementary Figure 3. Bar chart of clusters in each cancer stage in derivation, evaluation, and validation cohort.


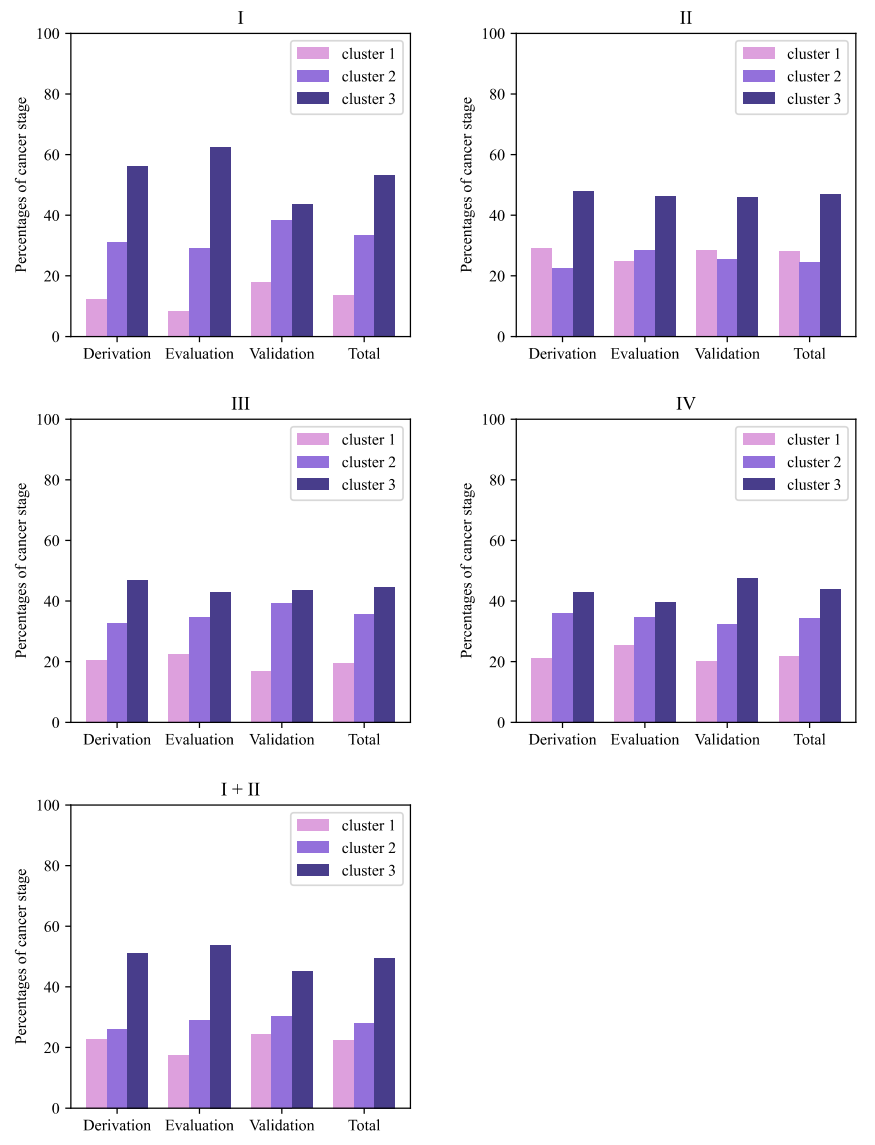


# Supplementary Figure 4. Violin plots of cancer classification score in different clusters in total cohort


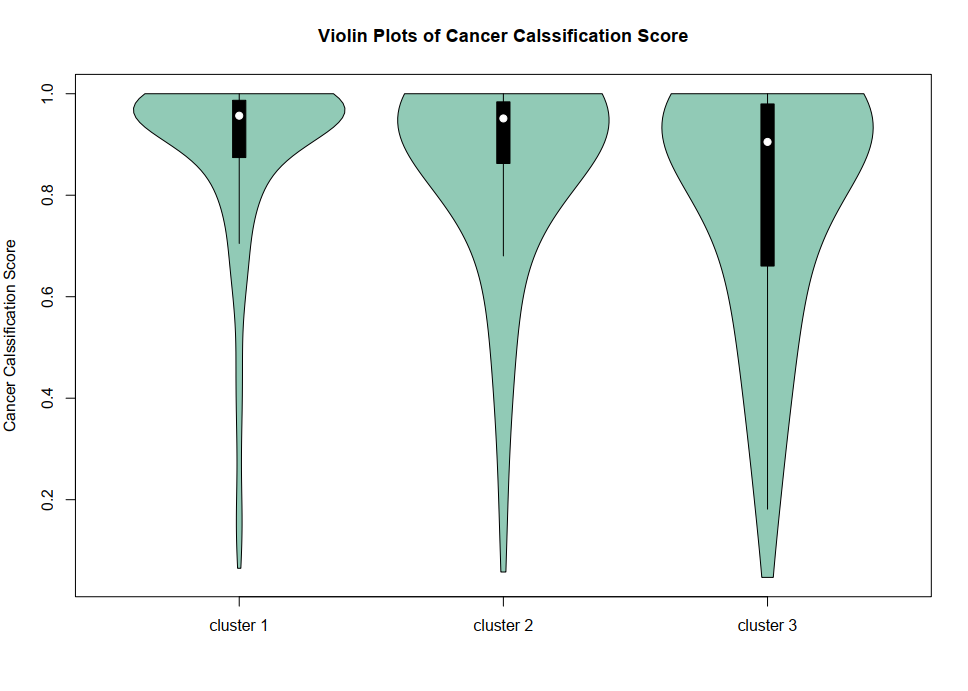


#
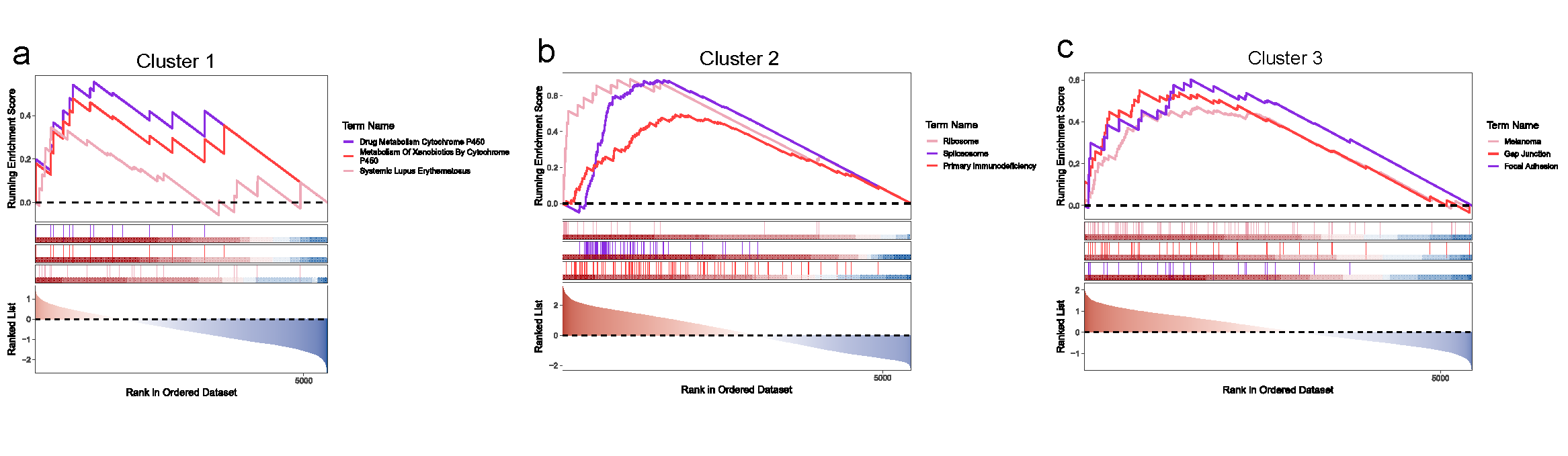
Supplementary Figure 5. Gene set enrichment analysis of each cluster in evaluation cohort based on KEGG.

Abbreviation: KEGG, Kyoto Encyclopedia of Genes and Genomes.

# **Supplementary Figure 6. Heatmap of expression of genes that participated in platelet activation and complement and coagulation cascades in three clusters.**


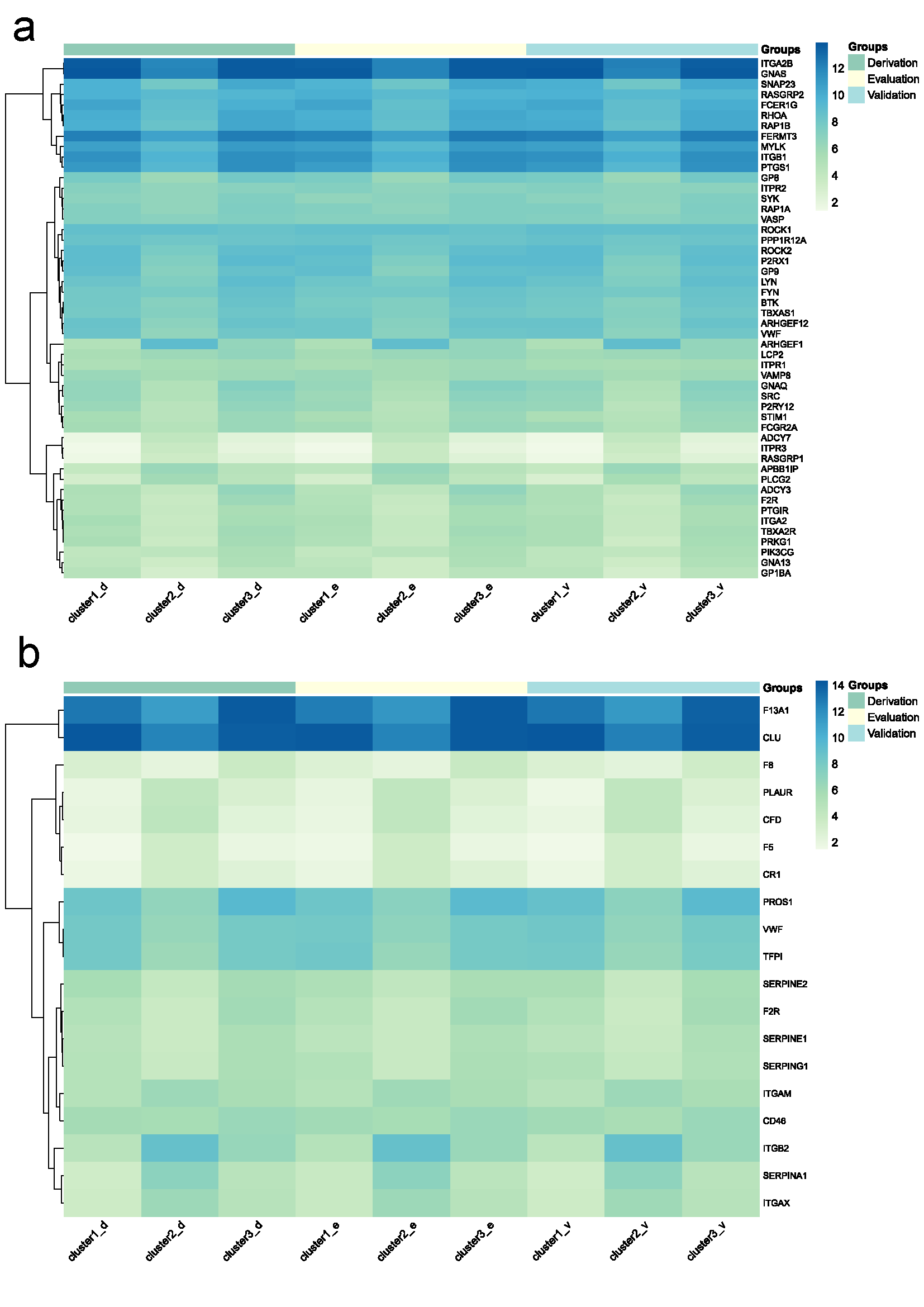


(a) platelet activation pathway; (b) complement and coagulation cascades pathway.

# **Supplementary Figure 7. Evaluation of potential effect of library size and age on prediction probabilities for 3 clusters.**


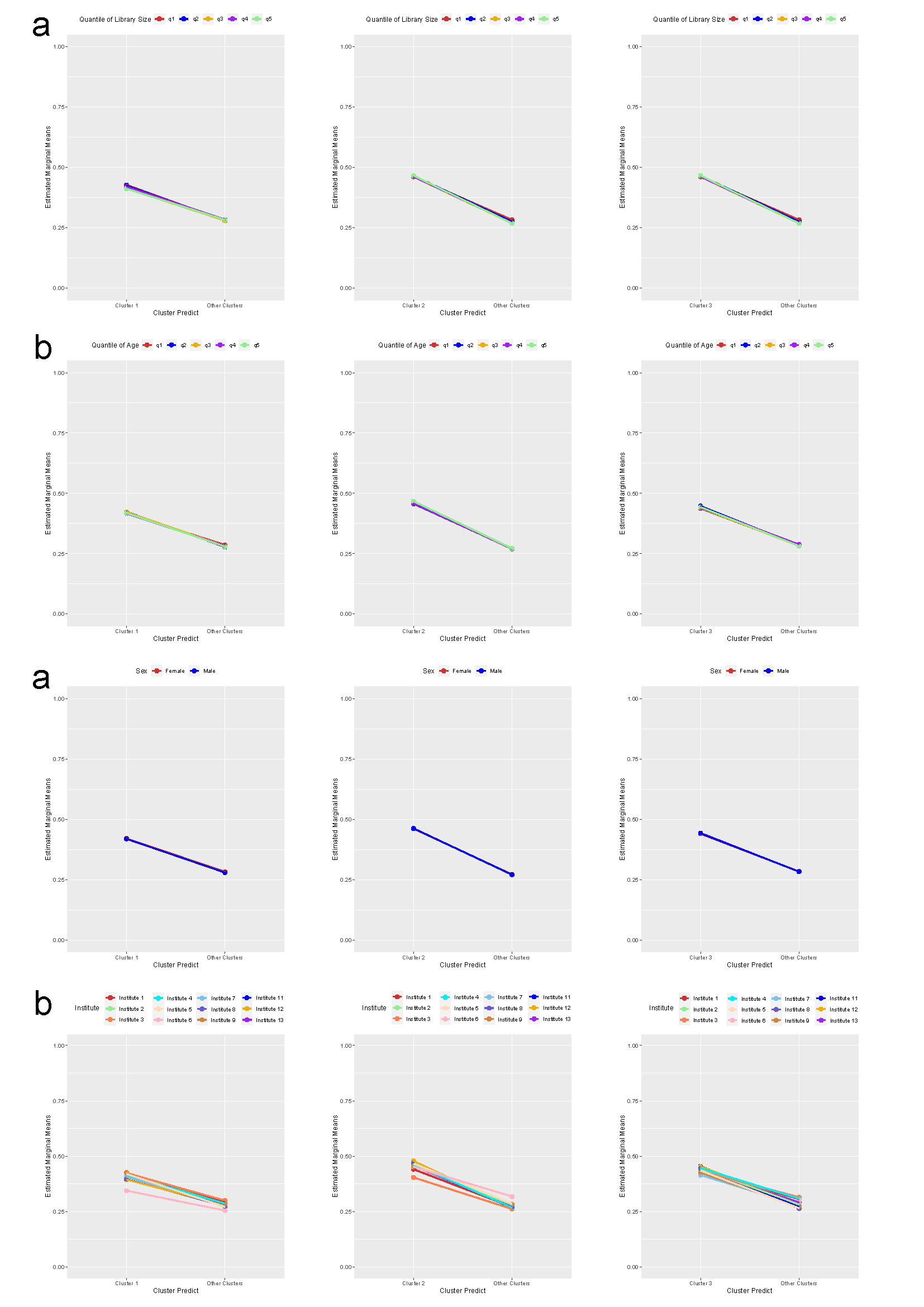


(a) Evaluation of library size. The library size was grouped into 5 groups based on five quantiles; (b) Evaluation of Age. The age was grouped into 5 groups based on five quantiles.

# **Supplementary Figure 8. Evaluation of potential effect of sex and institution on prediction probabilities for 3 clusters.**


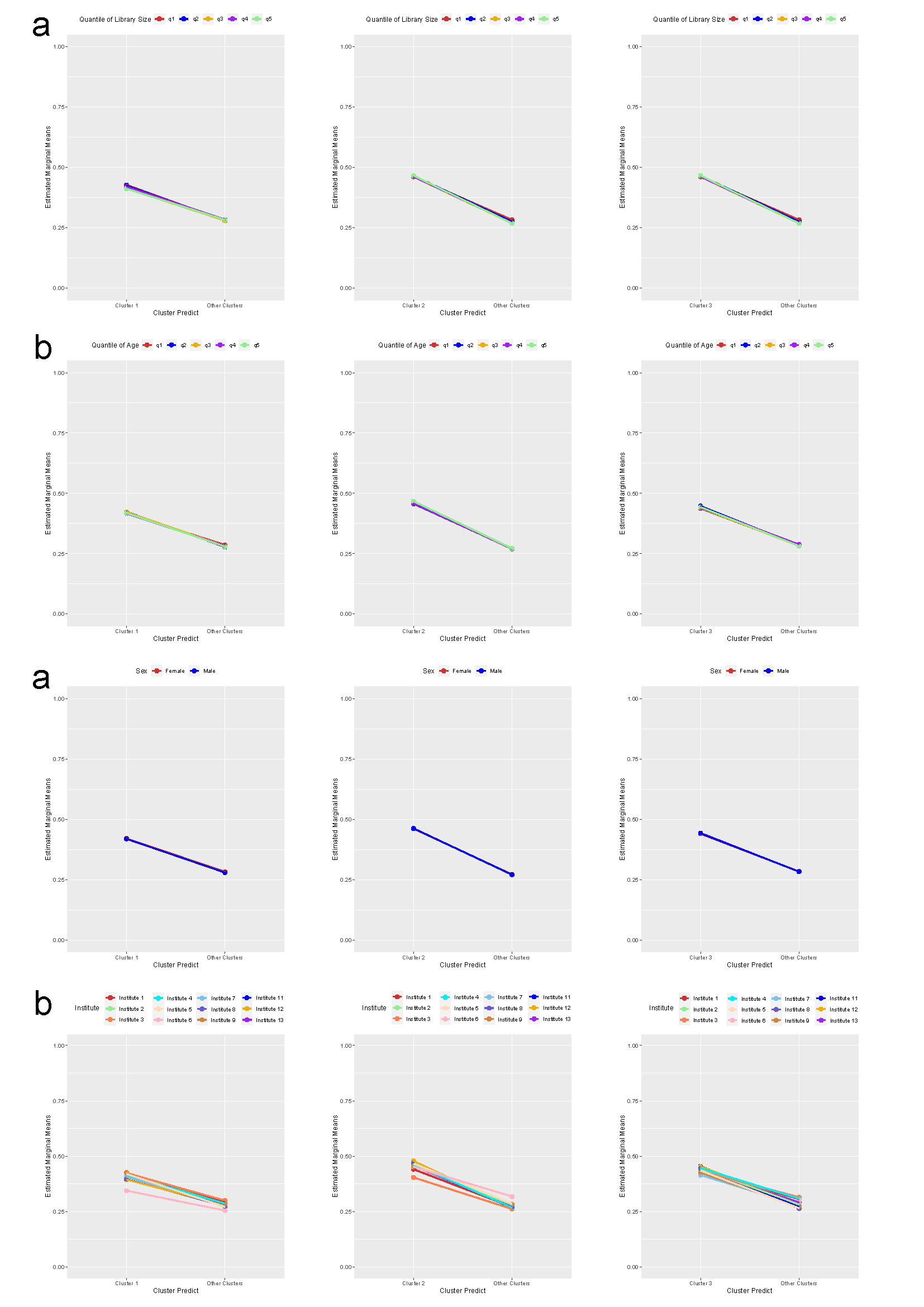


(a) Evaluation of sex.; (b) Evaluation of institution. Only institute 6 had group size ≤ 5.
