# Supplementary material for: Unsupervised and supervised machine learning to identify variability of tumor-educated platelets and association with pan-cancer: A cross-national study
Source: Fundam Res. 2023 Nov 2;6(1):498–508. doi: 10.1016/j.fmre.2023.09.004 (PMC12869750; doi:10.1016/j.fmre.2023.09.004)
Supplement: Supplementary file 2 [file mmc2.pdf]

ELSEVIER

Certificate of Elsevier Language Editing Services

The following article was edited by Elsevier Language Editing Services:

"Unsupervised and Supervised Machine Learning to Identify Heterogeneity of Tumor-Educated Platelets and Association with Pan-Cancer: A Cross-National Study"

Authored by:

Shaoxu Wu, Xiong Chen, Runnan Shen, Kai Huang

Date: 30-Dec-2022
Serial number: LE-254120-3CAB730F8C77
